# Molecular Interactions of Norfloxacin in Metal-Loaded Clay Suspensions-Effects on Degradation and Induced Toxicity

**DOI:** 10.3390/ijms27010459

**Published:** 2026-01-01

**Authors:** Roumaissa Djidja, David Dewez, Abdelkrim Azzouz

**Affiliations:** 1 Nanoqam, Department of Chemistry, University of Quebec at Montreal, Montréal, QC H3C 3P8, Canada; djidja.roumaissa@courrier.uqam.ca; 2 Station Expérimentale des Procédés Pilotes en Environnement (STEPPE), École de Technologie Supérieure, Montréal, QC H3C 1K3, Canada

**Keywords:** zero valent metals, norfloxacin, Montmorillonite, adsorption, *Lemna minor*, toxicity

## Abstract

The role of the metal valence state on the surface properties of metal-loaded clay minerals in the adsorption/oxidative degradation of an antibiotic was investigated. Transitional metal cations and their zero-valent counterparts such as Fe^0^, Ni^0^, Co^0^ and Cu^0^ supported on montmorillonite were comparatively investigated for their interactions during adsorption and toxicity tests of antibiotic norfloxacin (NOF). UV-Vis spectrophotometric and Fourier transform infrared (FTIR) spectroscopic analyses confirmed the involvement of the hydroxyl and carboxyl groups and/or piperazinyl nitrogen of NOF in the complexation with metal cations. Ecotoxicological assessment using aquatic plants *Lemna minor* showed that the metal cations reduce the bioavailability of the organic pollutant and that the zero-valent metals display higher toxicity due to their specific interaction with NOF and clay mineral surface. This evaluation will provide insights into potential environmental impacts of the co-occurrence of antibiotics and metals and will certainly contribute to correlating the safety of the water treatment by assessing the residual toxicity and its fluctuations.

## 1. Introduction

Antibiotics have become a significant environmental issue due to the persistent impact of the parent compound and/or its derivatives in various ecosystems [1]. For instance, antibiotics can induce toxic effects on growth and photosynthesis of plants [2] and depletion of microbial communities responsible for essential ecological functions [3] This disruption can also impact pathogenic bacteria such as enterotoxigenic *Escherichia coli* (ETEC), potentially altering host-microbiome interactions and exacerbating infections [4] in spite of the well-known plant strategy to combat the oxidative stress [5,6]. Due to their extensive use and slow biodegradability, fluoroquinolones (FQs) and more particularly norfloxacin (NOF) are interesting to be investigated in this regard [7,8,9]. The similarity of FQs with quinones confers norfloxacin (NOF) a similar action as a quinone site inhibitor in photosystem II (PSII) [10].

This impact should be governed by specific behavior and interactions of the organic substrate in clay-containing media and in even more in the presence of metal [11,12]. Here, these interactions are expected to unavoidably lead to the formation of complexes that can modify the reactivity towards oxidizing/reducing agents but the induced residual ecotoxicity. In this regard, research on FQs behavior combined with metals present in soils and aqueous media and its effect remains limited [8]. The co-occurrence of FQs and heavy metals in the environment may induce changes in their slow degradation and their specific toxicities. For instance, NOF and copper salts have been detected in livestock and poultry wastewaters and soils with concentrations reaching up to 225.45 ppm and 160 ppm, respectively [13,14]. The different natures of pollutants imply different behaviors in ecosystems [15] and possibly synergistic ecotoxicity on the environment and human health [16,17].

In natural media, antibiotics can undergo diverse degradation pathways, such as photolysis and photo-oxidation [18,19,20], aerobic/anaerobic bacteria-driven processes and ozonation on the outermost surfaces under severe or prolonged exposure to air and sun radiation. Ozonation already turned out to be quite effective in quinolone removal [9]. The pH [21], light exposure [22] and presence of reactive minerals [23] were found to play key-roles in this process. Among many minerals, clays dispersed in soils and waters are assumed to have a high capacity to retain and interact with organic molecules and metals regardless of their oxidation state with beneficial and/or detrimental effects on the polluted media. For example, bentonite-supported zero-valent iron showed effectiveness in the simultaneous removal of Cr(VI) and phenol [24]. Besides, clay mineral cation exchange is a conventional strategy for water remediation [25].

Due to their variable oxidation states, chelating ability, Lewis acid behavior, and redox properties transition metals can form stable complexes with organic drugs [26]. FQs can interact with the exchangeable cations of clay minerals through both ion exchange or complexation mechanisms. Ciprofloxacin (CIP) was found to easily adsorb on pillared bentonite in alkaline media [27] mainly through van der Waals interactions in spite the repulsive force of negatively charge CIP and clay mineral surface [28]. The ammonium and carboxyl groups of Norfloxacin can promote cation exchange and chelating processes that should strongly influence its mobility in soils and groundwater according to the pH and competitive interactions of dissolved metal cations [12]. Metal cations were already found to strongly interact with norfloxacin, influencing both its adsorption and oxidative degradation pathways [29,30]. Thus, the metal nature and valence are expected to modulate the strength and nature of [Metal:NOF] interactions and the evolution of their toxicities.

In anaerobic environments, zero-valent metals (ZVM) may promote the reduction of organic substrates and even their already oxidized derivatives. This was supported by the contribution of transitional metal particles in nitroaromatic reduction [31,32,33,34,35,36,37], dechlorination of chlorinated hydrocarbons [38], and even of trichloroethylene (TCE) when supported on bentonite [39]. This was explained in terms of electron transfer improvement by high amount of surface hydroxyl groups and a synergistic effect of Fe^2+^/Fe^3+^ and Cu^0^/Cu^2+^ redox cycles [40]. Here, the surface oxidation of ZVM generates metal oxides that promote ozone decomposition [41] but also Fe(II) cation release with additional potential toxicity [42,43]. This can be elucidated through deeper investigations. The beneficial role of surface hydroxyl groups was also reported for MnOx/SBA-15-catalyzed ozonation of NOF [44]. Such catalysts displayed fairly extended specific surface areas of 405–671 m^2^.g^−1^, affording however moderate but higher mineralization rate as compared to the non-catalytic ozonation. Furthermore, the presence of chelating sites such as hydroxyl groups is an essential requirement not only for preventing particle agglomeration and loss in catalytic surface [45], but also for promoting additional adsorption contribution for complete remediation [9].

Given the high number of factors that influence the heterogeneous catalytic process, adsorption resulting toxicity, the present research was completed by factorial experiment designs, a judicious approach for assessing the simultaneous individual effects and interactions [46] around supposedly optimum values of much lower number of parameters [47]. Similar approaches were already tackled for maximizing the adsorption capacity of ciprofloxacin [48], tetracycline [47] and norfloxacin on various materials [49]. In the present work, the pH, contact time, and clay catalyst concentration were considered as key factors in the retention, degradation and toxicity of NOF in metal-containing aqueous clay suspensions. This is intended not only to understand their complex interactions in natural clay-containing media such as soils and clay suspensions in ponds, lakes and rivers but also to explain toxicity evolution using *Lemna minor* as an aquatic plant bioindicator.

## 2. Results and Discussion

### 2.1. Metal Cation: NOF Interaction

**Figure 1:**
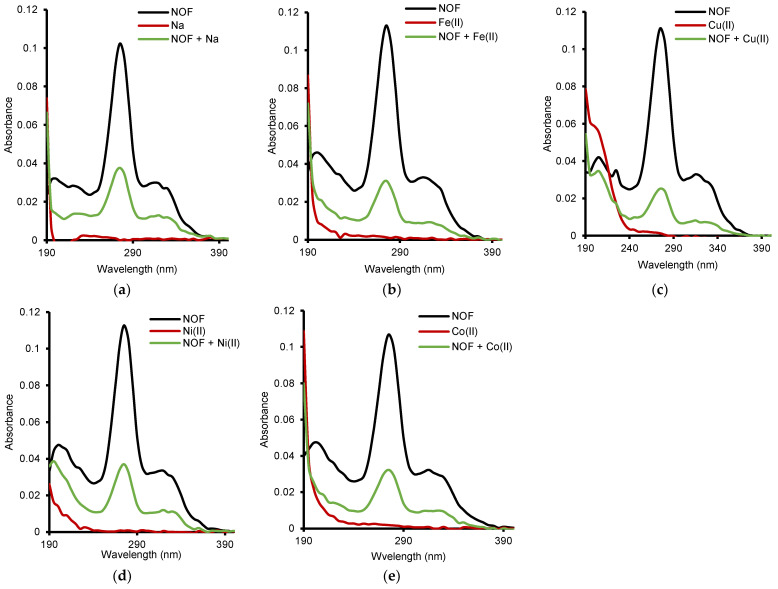
UV–Vis absorption spectra for Norfloxacin in the presence of Na^+^ (**a**), Fe^2+^ (**b**), Cu^2+^ (**c**), Ni^2+^ (**d**) and Co^2+^ (**e**). NOF concentration: 3.31 × 10^−6^ M. Metal cation concentration: 10 ppm.

**Scheme 1:**
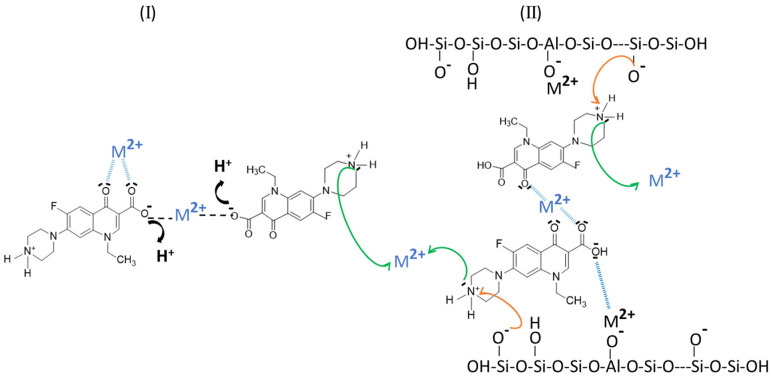
Potential NOF interactions with metal cation (**I**) and with metal cation and clay mineral surface (**II**). Orange: NOF ion exchange on deprotonated silanols; Green: NOF capture by adsorbed and free cation; Blue: Cation capture by chelation on NOF; Black: Cation capture by NOF though ion exchange. Part I describes i. the formation of NOF:NOF bridges via bivalent cation exchange on two deprotonated carboxyls of two free NOF molecules and ii. Lewis acid-base interaction between the metal cation and non-binding electron pairs of oxygen atoms (and potentially of N atoms also). Part II illustrates additional interactions involving NOF adsorption through exchange of i. ammonium cation on the clay mineral surface and ii. the formation of NOF-Clay bridges between NOF carboxyls and exchangeable sites sharing a bivalent metal cation.

NOF interaction with metal cations was reflected by noticeable changes in the UV-Vis spectrum of their mixture (Figure 1). The marked decrease in absorbance intensity at 275 nm and to a lesser extent at 315 nm indicates a depletion of free NOF molecules, which appears to evolve in time (Figure S1). This must be likely due to cation exchange on deprotonated carboxylic groups and complexation with free electron pairs of the carbonyl, ammonium and piperazine groups of NOF (Scheme 1). Here, the piperazine ring is assumed to be much less involved in the cation complexation, as already reported for fluoroquinolones with transition metals [50].

**Figure 2:**
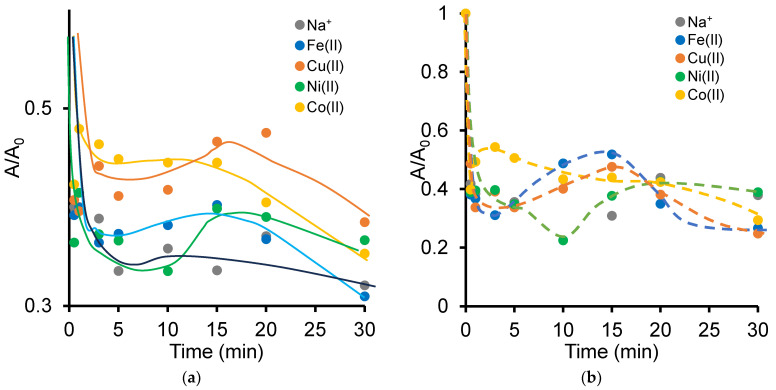
Evolution of the relative absorbance at 275 nm (**a**) 315 nm (**b**) during metal:NOF complex formation. The relative absorbance corresponds to A/A_0_, where A is the absorbance of NOF in the presence of metal ions and A_0_ is the absorbance of the initial NOF solution without metal with a standard deviation of ±0.04.

Such [Metal:NOF] interactions may contribute to the chemical stability of the antibiotic molecule, as supported by an increased photostability of [Cu^2+^-ciprofloxacin] system [51]. The pronounced decrease in intensity during the first 5–10 min followed by a bump between 5 and 30 min is a precise indicator of the occurrence of successive interactions involving slow ion-exchange and/or chelation processes (Figure 2).

Deeper insights through FTIR analysis allowed explain *i*. the involvement of the carboxyl and carbonyl groups in the cation capture, *ii*. the cation interaction with the N-H group of the imino-moiety of piperazinyl groups and/or the O-H of the carboxylic groups and *iii*. those occurring between the zero-valent metal and surface hydroxyl groups. The total disappearance of the C=O bond stretching around 1740 cm^−1^ confirmed that the carboxyl and carbonyl groups are involved in the cation capture (Figure S2). Cation addition to NOF also induced a significant intensity decrease and a slight shift of the broad band around 3260 cm^−1^ towards higher wavenumber of up to 3270 cm^−1^ for [NOF:Na^+^], 3370 cm^−1^ for [NOF:Fe^2+^], 3380 cm^−1^ for [NOF:Cu^2+^], 3280 for [NOF:Ni^2+^] and 3320 cm^−1^ for [NOF:Co^2+^] suspected interactions (Table S5). This can be attributed to cation interaction with the N-H group of the imino-moiety of piperazinyl groups and/or the O-H of the carboxylic group, in agreement with the previous statement. These FTIR band shifts were assigned to the NH- and OH bond stretching and appear to be stronger for Fe^2+^, Cu^2+^ and Co^2+^ as compared to Ni^2+^ and to a lesser extent Na^+^. This indicates the occurrence of different interaction strengths of the corresponding groups according to the metal cation, which remains to be confirmed by X-ray photo-electron spectroscopy which provides an accurate assessment of the slight variations of the binding energy of the electrons belonging to the chelating groups [4,52]. In the meantime, new signals were observed at 1620–1670 cm^−1^ attributed to N-H bending vibration of the quinolone group [21,50,53].

These interactions are expected not only to drive metal cation transfers between the bult solution, NOF molecules and clay mineral surface. These wavelength shifts should, in turn, govern NOF adsorption in the forms of free molecules, metal cation/NOF salts and [NOF:Cation] complexes on the clay mineral surface but also the dispersion of the resulting NOF-loaded clay mineral lamellae.

### 2.2. Effect of [NOF:Metal] Interaction on Clay Mineral Dispersion During Adsorption

In the presence of Na^+^-montmorillonite (NaMt) and its and bivalent metal cation exchanged counterparts (Fe(II)Mt, Co(II)Mt, Ni(II)Mt and Cu(II)Mt), it appears that both adsorption and ozonation induce change in the chemical composition of the aqueous media as observed through periodical ICP-OES analysis in time.

**Figure 3:**
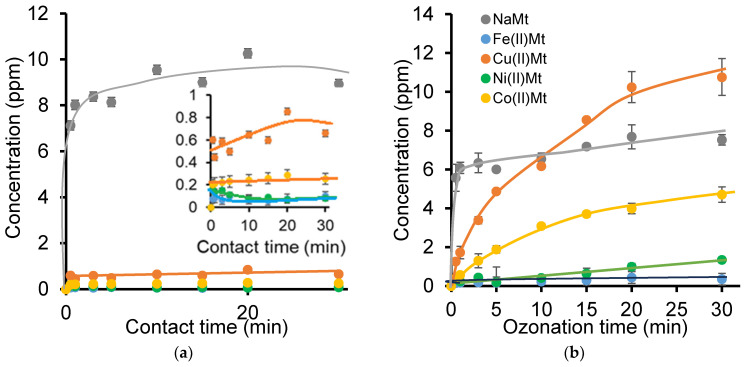
Evolution of the metal cation concentration in the NOF solution during adsorption (**a**) and clay-catalyzed ozonation (**b**). T = 25 °C. pH = intrinsic level. Clay mineral amount: 50 mg. Sample volume: 25 mL. NOF initial concentration = 3.31 × 10^−6^ M.

Increasing contact time during adsorption induced a pronounced Na^+^ release in the liquid media (Figure 3a). This is presumably due to a relatively strong cation exchanged imposed by the low initial pH (5.2) of the starting NOF solution with respect to the basic character of Na^+^ [30]. This low pH should not produce noticeable release of transition metal cations in the bulk solution, as reflected through their barely detected amounts by ICP-OES. In contrast, the ozonation progress in time led to a continuous increase of the concentration of dissolved transition metal cation, except for Na^+^ which rapidly reached almost the same level as during adsorption. The latter was slightly mitigated after only 1–2 min contact time probably due to interaction with NOF and rise of acidic NOF derivatives (Figure 3b).

**Figure 4:**
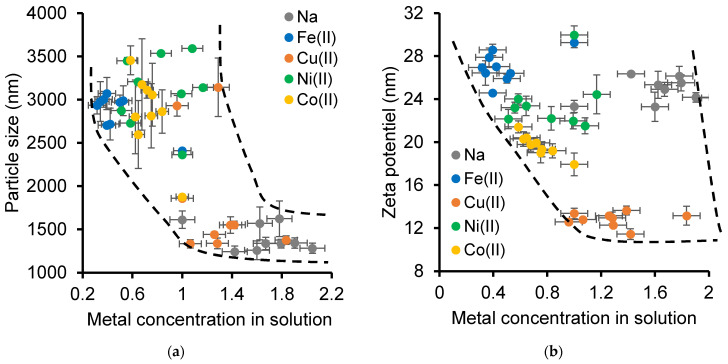
Particle size (**a**) and zeta potential (**b**) as function of the fraction of free metal ions detected in NOF solution during adsorption. T = 25 °C. NOF solution: initial pH = 5.2. Clay mineral amount: 50 mg. Sample volume: 25 mL. NOF initial concentration = 3.31 × 10^−6^ M.

The most plausible explanation resides in a progressive cation release through ion-exchange promoted by the graduation formation of acidic species and pH decrease. This cation release was found to decrease in the following sequence Cu(II) > Co(II) > Ni(II) > Fe(II). Attempts to correlate the effect of this cation release in the bulk solution with the clay mineral dispersion gave only a global decrease in the particle size with increasing amount of cation detected (Figure 4a). The finest particle sizes were observed around 1200–1700 nm for in the presence of dissolved Cu^2+^ and Na^+^, while the bulkiest ones were noticed for Ni^2+^, Co^2+^ and Fe^2+^ cations.

This cannot be explained only by the decreasing polarizing power and Lewis acidity of the transitional cations in the sequence Ni(II) > Cu(II) > Co(II) > Fe(II). Here, the unavoidable effect of the presence of NOF molecules and competitive interactions with both forms of dissolved and adsorbed cations must also be involved, in agreement with Scheme 1. The particle size decrease contrasts with the decrease in the Zeta potential (ZP) and increase in the amount of free metal cation, which should rather trigger progressive surface protonation and clay lamella aggregation and compaction (Figure 4b). This suggests that increased amount of released cation should globally produce an acidity improvement that promotes the adsorption of positively charged NOF species through their protonated ammonium groups, without necessarily leading to clay mineral aggregation.

At this pH level, NOF occurs predominately as a protonated species that adsorbs on the clay mineral surface via electrostatic interactions and simultaneously chelates both free and adsorbed metal cations. The most common coordination sites include the carboxylic and ketone groups. In addition, nitrogen atom of the piperazine ring and, in some cases hydroxyl atoms can also participate in coordination [21,51,54]. The involvement of these sites depends on factors such as pH, metal cation properties, and ligand-to-metal ratio [55]. Possible interactions between NOF molecules and the clay mineral surface may involve mainly hydrogen bonding and electrostatic forces. The metal ion can coordinate with NOF either in a 1:1 (I) or 1:2 (II) (Metal:Ligand) ratio depending on the metal type, pH, and experimental conditions. Some studies, based on the Lineweaver–Burk equation, indicates that Cu(II) can form 1:1 complexes with enrofloxacin [56]. That is why pH variation during ozonation plays a crucial role in controlling all binary [NOF:Cation]. [NOF:NOF], [Clay:Clay] and [Clay:Cation] and ternary [NOF:Clay:Cation] and [Clay:NOF:Cation] interactions through protonation/deprotonation of the functional groups involved [30].

Montmorillonite shows adsorption capacity that vary according to the acid-base character of the exchangeable cation and the charges of NOF molecules. NOF retention involves pH-dependent interactions that strongly influence the clay mineral aggregation/dispersion and the extent of the adsorption surface. At a intrinsic pH of the 1 mg/L NOF solution, Fe(II)Mt and Ni(II)Mt gave a 100% NOF removal rate (0.5 mg/g), versus 99–98% with Cu(II)Mt (0.47 mg/g) and barely 55–60% with NaMt (0.275–0.30 mg/g) [30]. These values vary according the pH and initial NOF concentration, which should be close to those of most NOF-contaminated wastewaters, and are in the same magnitude order as those provided by the literature under nearly similar conditions [57,58]. These different values appear to strongly influence the degradation of NOF and induced toxicity towards *L. minor*, which is the main objective of the present research. Nevertheless, the use of montmorillonite as adsorbent for NOF removal purposes often require improvements of the hydrophobic interaction by incorporating organic moiety as reported by ample literature.

### 2.3. Clay Mineral Amount Effect on Ozonation and Toxicity

**Figure 5:**
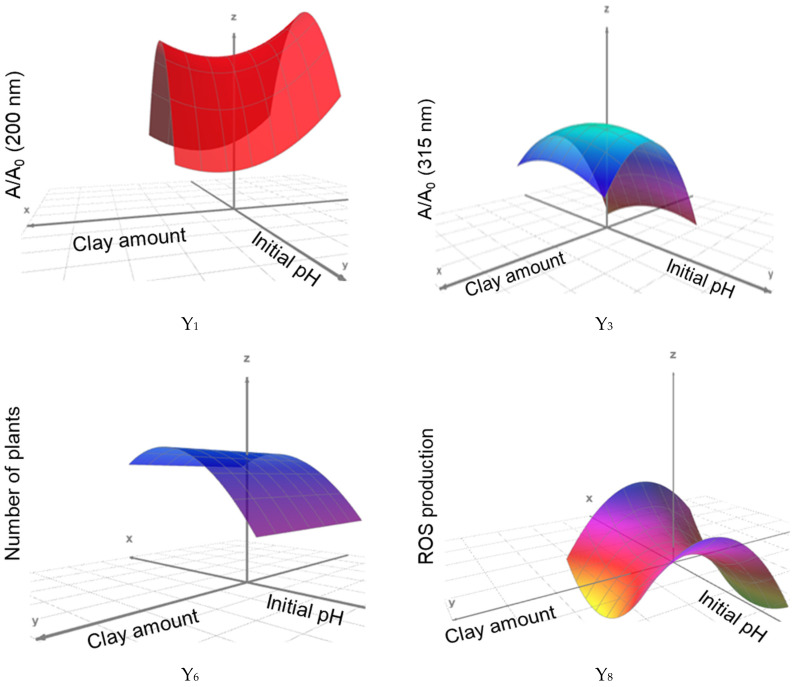
Response-surface of NOF ozonation progress as expressed in terms of A/A_0_ at 200 nm (Y_1_) and 315 nm (Y_3_) for Fe(II)Mt and central reaction time (X_3_ = 0) and toxicity as expressed by the number of plant specimens (Y_6_) and ROS production (Y_8_).

Previous works already demonstrated the key role of clay minerals on the ozonation of organic substrates [29,30,59,60,61,62], but only few were devoted to such interaction, more particularly in correlation with the role of the catalyst amount on adsorption [59,63,64]. In this regard, correlating the effects of pH and ozonation time to the catalyst amount could be a judicious approach for assessing the optimum parameter effects for maximum removal of NOF and minimum toxicity. For this purpose, an given the high number of parameters and metal cations, a comparative study of ozonation through two 3^3^ factorial experiment designs was restricted to only NaMt and Fe(II)Mt as the catalysts. In this regard, 27 ozonation attempts were performed for each clay catalyst within judiciously established variation ranges (Table S6) and model coefficient calculations (Table S7) revealed a favorable individual effect of pH in presence of NaMt for characteristic UV bands of NOF (Y_1_, Y_2_ and Y_3_). The sign of the coefficient describes the effect nature while the absolute value of the coefficient expresses the effect intensity [59,65,66,67,68]. The mere occurrence of optimum values of the pH, clay mineral concentration and ozonation time with optimum productions of NOF intermediates absorbing at the three wavelengths and of optimum values of the toxicity biomarkers considered (Table S8). This provides clear evidence of the occurrence of optimum favorable interaction, more particularly of the initial pH and clay mineral amount, for maximum NOF degradation in presence of NaMt and Fe(II)Mt and minimum induced toxicity (Figure 5).

As a general feature, increase in clay amount influences NOF ozonation (Y_2_ and Y_3_), which, in turn, determine the optimum pH for maximum clay mineral dispersion and contact surface according to the Critical Micellar Concentration of montmorillonite [67]. This also impacts the ratio chl *a/b* (Y_7_). NOF conversion increase in time in presence of Fe(II)Mt (a_3_ = 14.786) more than NaMt (a_3_ = 4.965). As the ozonation process progresses, the production of reactive oxygen species (ROS) increases (Y_8_) especially with Fe(II)Mt. The most significant interaction was registered for NOF toxicity (a_13_ = 233.395) through the initial pH and ozonation time. This indicates a synergy of these two parameters on ROS level (Y_8_) with Fe(II)Mt. Strong interactions were also noticed between the pH and clay catalyst (a_12_) for NOF conversion in presence of NaMt (Y_1_, Y_2_, Y_3_). pH fluctuations are expected to impact the catalyst activity, and conversely, as already reported for the ozonation of oxalic acid with different catalyst [59] and Orange-G with hematite/SBA-16 [62].

A favorable interaction for the three parameters with NaMt for the formation of hydroxylated and acidic intermediates was noticed for the relative absorbance (A/A_0_) at 200 nm (Y_1_) and ROS production (Y_8_). This indicates the formation of intermediates with high toxicity towards *L. minor*, which contrasts with Fe(II)Mt where a positive a_123_ coefficient was obtained for fresh weight and fronds number (Y_4_ et Y_5_). This result is of great importance, because it demonstrates that an increase in initial pH, clay mineral amount and ozonation time gave rise to less toxic end products.

### 2.4. Ozonation Effect on Photosynthetic System

**Figure 6:**
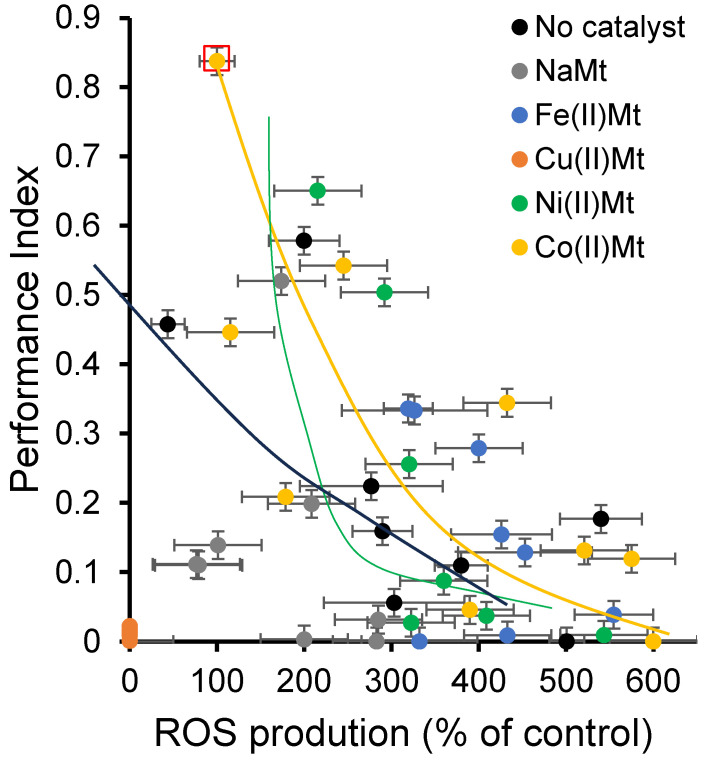
Correlation between the performance index of the photosynthetic system (PI_ABS_) and ROS production of *Lemna minor* exposed to clay-catalyzed ozonation of norfloxacin. The red square accounts for the control sample used for ROS normalization. ROS are expressed as a percentage with respect to this control, which showed the highest performance index. The orange points represent the values assessed for the copper-containing samples. All these values are close to zero due to an almost total plant mortality.

Clay-catalyzed ozonation of NOF induced changes in the frond number, fresh weight, chlorophyll content and ROS production by *Lemna minor* indicating alteration in the pigment composition of the photosynthetic apparatus [29] but its influence on photosynthetic activity remains unexplored. In photosynthesis, photosystem II defined as an enzyme complex located in the thylakoid membranes of chloroplasts [69]. Deeper analysis of photosynthetic efficiency was carried out focusing on PSII performance. The altered OJIP transients based on a fluorescence induction curve plotted on a logarithmic time scale to describe the PSII electron transport activity (Figure S3) revealed a progressive decline in the photosynthetic performance following exposure *Lemna minor* to ozonated NOF solutions. A correlation between PI_ABS_ and production of ROS showed a general tendency (with few exceptions) consisting in decrease in photosynthetic performance is accompanied by an increased oxidative stress (Figure 6).

**Figure 7:**
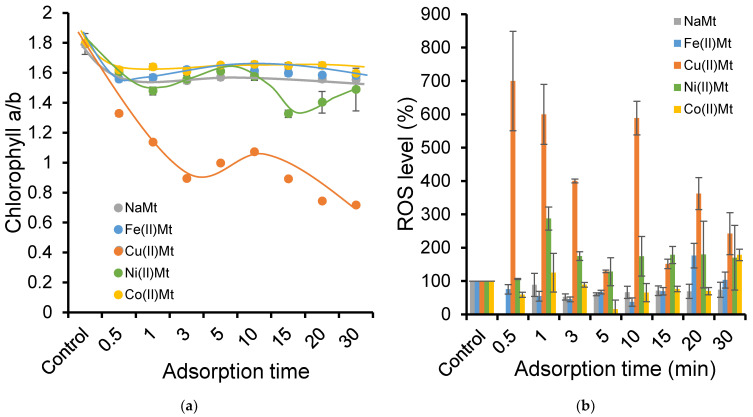
Effect of combined NOF and metal ion on chlorophyll *a/b* ratio (**a**) and ROS level (**b**) after 7 days of exposure to *Lemna minor.* Triplicates measurements were achieved including the control sample. The control sample consisted of plants grown in SIS medium.

FQs such as CIP disrupt the energy transfer between antenna chlorophyll molecules and the reaction center of photosystem II. This disruption leads to an accumulation of non-functional excited chlorophylls, a slower photoreduction of the primary quinone acceptor Q_A_ and consequently a reduction in the overall photochemical efficiency of PSII [70]. FQs such as NOF were already found to exhibit a similar effect, but this toxicity appears to be enhanced by the presence of metal cations [30]. Ozonation was found to trigger a progressive release of metal ions revealed by ICP-OES analysis in the liquid medium from the clay mineral (Figure 3b). This arises from the progressive pH decrease due to the rise of acidic derivatives during ozonation, inducing cation exchange and release in the aqueous media by a proton excess. This explains, at least partly, the ozonation mitigation time, imposing periodical catalyst regeneration by reverse cation exchange [59]. The consecutive accumulation of metal ions in the SIS medium (Figure S4a–e) is expected to result in higher contents in plant tissues. Cu(II) and Co(II) showed marked increases in their concentrations during the ozonation process (Figure S4c,e), confirming their high bioavailability and potential involvement in metal–pollutant complexes taken up by the plants. Once accumulated in plant tissues, metals cations can promote oxidative stress by generating reactive oxygen species (ROS). Moreover, metal ions can interact directly with chlorophyll molecules or photosynthetic enzymes, altering their structure and function. Based on these results, the toxic impact of clay-catalyzed ozonation of norfloxacin was stronger on the PSII activity of *L. minor*, due to a higher intracellular accumulation of metals and/or norfloxacin and its transformation products. Simultaneous exposure *Lemna minor* to NOF and metal ions reduced the growth parameters such as frond number and fresh weight (Figure S5). This combination also affected the chlorophyll content and ROS accumulation (Figure 7).

### 2.5. NOF Interaction with Clay-Supported Zero-Valent Metal

The incorporation of ZVMs into montmorillonite induced a red shift of the broad FTIR band around 3500 cm^−1^ (Figure S6). This band is assigned to O-H stretching vibrations [71]. This shift toward lower wavenumbers indicates interactions between ZVMs and surface hydroxyl groups of the clay mineral likely through coordination or hydrogen bonding. For Cu^0^Mt, the new band at 1412 cm^−1^ confirmed the immobilization or reduction of Cu^0^ [72].

**Figure 8:**
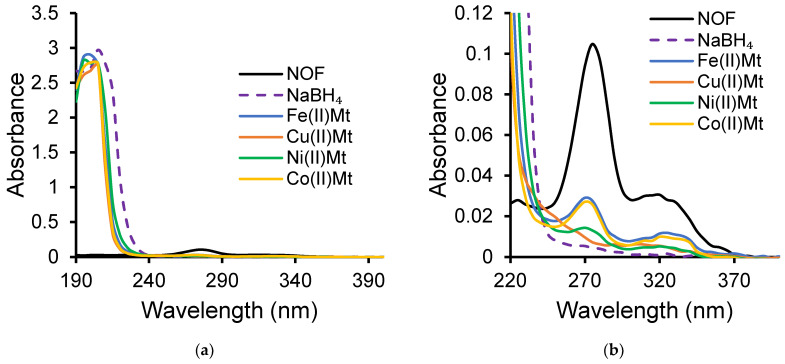
UV–Vis Spectra of NOF-Metal exchanged montmorillonite mixtures upon NaBH_4_ reduction under nitrogen atmosphere (**a**) and close-up for the region 220–370 nm (**b**) as compared to NOF alone, NOF + NaBH_4_ mixture and NaBH_4_ alone. Clay mineral amount: 50 mg. Sample volume: 25 mL. NOF initial concentration = 3.31 × 10^−6^ M. 10 mL NaBH_4_ (0.1 M).

M^0^ incorporation in clay mineral particles is expected to contribute to surface interactions and possible complexation with both free and adsorbed NOF molecules. The intensity of the absorbance of the characteristic UV-Vis band of NOF at 275 nm (Figure 8) rapidly decreased up to total disappearance upon direct contact with M^0^/Mt, suggesting strong NOF interaction with the surfaces of the metal-loaded clay minerals and advanced depletion of free NOF molecules.

**Figure 9:**
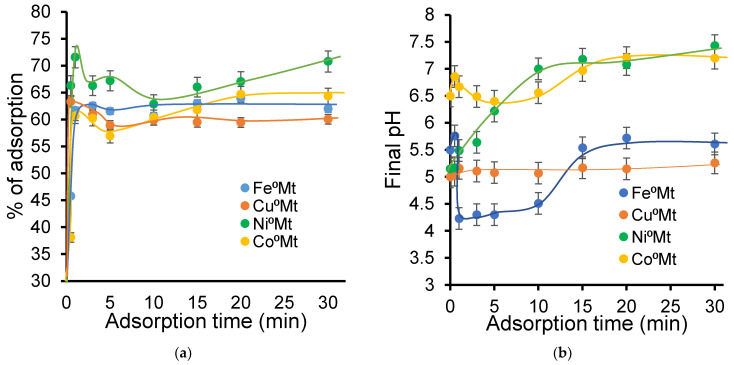
Evolution in time of adsorption yield as determined by HPLC-DAD (**a**) and evolution of instant pH (**b**). Clay mineral amount: 50 mg. Sample volume: 25 mL. NOF initial concentration = 3.31 × 10^−6^ M.

This was confirmed by HPLC analysis, which showed a ca. 60–70% reduction in the NOF peak area upon NaBH_4_ addition, slightly higher in presence of Ni^0^Mt and slightly lower for Co^0^Mt (Figure 9a). This provides clear evidence of the reduction of transitional cation on the montmorillonite surface. The disappearance of the cationic species which commonly induce acidic pH agrees with the visible pH increase during the metal reduction process (Figure 9b).

Cu^0^Mt displays almost similar adsorptive and catalytic properties with almost similar effects as Fe^0^Mt on the biomarkers that allow assessing NOF toxicity towards *L. minor*. Nevertheless, copper is known to be a more toxic metal in both cationic and zero-valent forms as compared to iron, which can rather act as nutrient for plants in suitable concentrations.

### 2.6. Toxicity of NOF Adsorbed on ZVM-Loaded Montmorillonite

**Figure 10:**
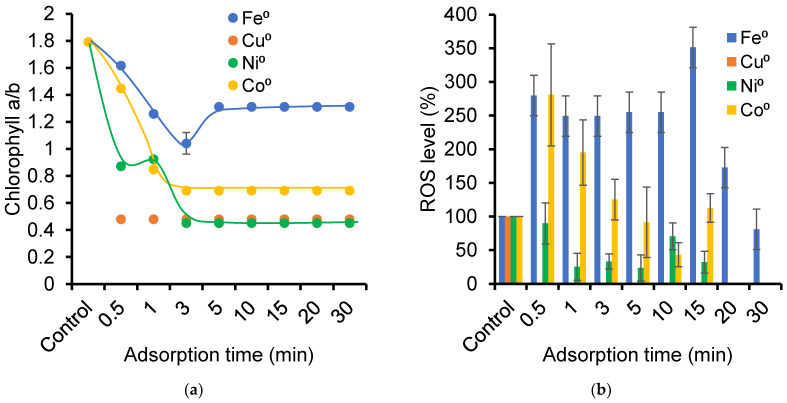
Change in Chlorophyll ratio (**a**) and ROS level (**b**) of *Lemna minor* in NOF adsorption in clay-supported ZVM after 7 days of exposure. Triplicates measurements were achieved including the control sample. The control sample consisted of plants grown in pure SIS medium.

As compared to the starting exchanged montmorillonite M(II)Mt, ZVM-loaded montmorillonite (M^0^Mt) exhibited higher toxicity towards *L. minor* (Figure 7). After 30 min of exposure, the Chl *a/b* ratio decreased from 0.4 for Cu^0^Mt compared to 0.7 for Cu(II)Mt, from 1.5 for Ni(II)Mt and Co(II) to 0.45 for Ni^0^/Mt and 0.7 for Co^0^/Mt, and from 1.6 for Fe(II)Mt to 1.50 for Fe^0^Mt (Figure 7a and Figure 10a). The most pronounced reduction was observed for nickel, where the Chl a/b ratio dropped by approximately 70%, followed by copper with 43%, cobalt with a 53% decrease. Iron showed only a slight reduction of 6%. M^0^/Mt induced a decrease in the chlorophyll a/b ratio (Figure 10a), while Fe^0^/Mt and Co^0^/Mt gave higher ROS production as compared to the control sample (Figure 10b). In the meantime, a strong plant growth inhibition was observed, as reflected by a decrease in both frond number and fresh weight (Figure S7).

This difference is explained by the distinct mechanisms involved in each system. In the case of M(II)Mt, the toxicity involves ionic interactions through ions that can bind to cellular components but are partially regulated by physiological defense mechanisms into lower concentration [30]. Conversely, M^0^Mt undergoes gradual surface oxidation with the production of reactive oxygen species more particularly with water and dissolved [73], which amplifies the overall toxic response. ZVM were already used for the aerobic removal of dyes contaminants methyl orange and congo red at neutral pH, involving Cu^0^ reduction into Cu(I) and formation of hydroxyl radicals [74,75]. Reduction appears as being less efficient than oxidation, given that ciprofloxacin removal, for instance, was about 100% in presence of oxygen and 35% in anaerobic conditions after 2 h of reaction [76]. Here, Cu^0^ is assumed to oxidize into Cu(I) and Cu(II) releasing electrons that reduce oxygen and generate ROS, which are responsible for the CIP degradation.

However, under oxygen-free conditions and the presence of a reducing agent, zero-valent iron (Fe^0^) is assumed to react with protons and/or dissolved oxidizing agent in water, releasing electrons and consuming protons to generate highly reducing active hydrogen species [77]. These active hydrogen atoms subsequently donate electrons to organic cations, with potential cleavage of -C=N and -C=C bonds. ZMV were recognized as being highly effective for the aerobic degradation of azo dyes, but usually show lower efficiency in non-azo organic pollutants such as rhodamine B, methylene blue, and sodium pentachlorophenate [74]. The presence or absence of oxygen determines whether pollutant removal is dominated by oxidative/coagulation processes or by reductive transformations.

## 3. Methods and Materials

### 3.1. Chemicals and Stock Solutions

Norfloxacin (Lot No. LRAD2292) and Sodium tetrahydroborate (NaBH_4_) were purchased from Sigma Aldrich. Metallic salts FeCl_2_∙4H_2_O, NiSO_4_∙6H_2_O, CoCl_2_∙7H_2_O were supplied by Fisher Scientific, Pittsburgh, PA, USA (purity of 99%) and CuSO_4_.5H_2_O supplied by Sigma-Aldrich, Burlington, MA, USA (purity of 99.995%). Stock solutions of NOF (10 mg/L) were prepared in nanopure water and stored at 4 °C in the dark. Metals stock solutions (100 mg/L) were also prepared in nanopure water and stored under the same conditions.

### 3.2. Preparation of Montmorillonite-Based Adsorbent/Catalysts

Ion-exchanged montmorillonites Fe(II)Mt, Ni(II)Mt, Co(II)Mt, and Cu(II)Mt were prepared by impregnating NaMt with aqueous solutions of the corresponding metal salts following a previously described procedure [29,30]. Zero-valent metal modified montmorillonites (Fe^0^Mt, Ni^0^Mt, Co^0^Mt, and Cu^0^Mt) were prepared by dispersing 0.2 g of NaMt in 15 mL of 0.12 M aqueous solutions of the corresponding metal salts and stirred vigorously for 2 h. Then, 10 mL of NaBH_4_ (0.2 M) was added dropwise as a reducing agent under a nitrogen atmosphere, and the mixture was stirred for an additional 15 min. The color of the mixture turned dark, indicating the formation of ZVM. The resulting M^o^/Mt were then filtered, washed and dried for 3 h at 80 °C under N_2_ atmosphere and then stored in a sealed enclosure with dry oxygen-free nitrogen [78,79]. M^o^/Mt were characterized by Attenuated total reflectance-Fourier transform infrared spectroscopy, ATR-FTIR (Thermo Scientific, Nicolet 6700 instrument, Madison, WI, USA).

### 3.3. Metal-NOF Interaction Study

Prior to NOF adsorption and ozonation tests, the potential interaction of norfloxacin (NOF) in aqueous solution (3.31 × 10^−6^ M) with aqueous media containing various zero-valent metal and cation was investigated. The mixtures were stirred continuously at room temperature and was monitored by UV–Vis spectrophotometry using an Agilent-Cary 60 instrument and a 1 cm quartz cell and analyzed trough ATR-FTIR. A few drops of the aqueous complex sample were previously deposited directly onto the ATR crystal then air-dried at room temperature until complete evaporation.

### 3.4. Adsorption Tests

Adsorption experiments were carried out by contacting 50 mg of metal-exchanged montmorillonites (M^2+^-Mt) and zero-valent metal-modified montmorillonites (M^0^/Mt) with 25 mL of NOF solution at intrinsic pH. The suspensions were vigorously stirred at room temperature. The samples were analyzed immediately after filtration through a 0.45 mm membrane. The residual concentration of NOF in the supernatant was measured by UV–Vis spectrophotometry at 275 nm and High-Performance Liquid Chromatography coupled to a Diode Array Detector detector (HPLC-DAD, Agilent Technologies model 1290 equipment) under specific conditions (Table S1). The adsorption yield was defined as (1 − (A/A_0_)) × 100%, where A is the instant HPLC-DAD peak area and A_0_ the initial HPLC-DAD peak area.

Zeta potential (ZP) and particle size variation was measured during adsorption through a Malvern Zetasizer device Ultra Red Label (Malvern Panalytical, UK). The concentration of metal cations released from the clay minerals into the solution was determined after each adsorption or ozonation test using Inductively Coupled Plasma Optical Emission Spectrometer (ICP-OES, Agilent 5100, Santa Clara, CA, USA), with an axial plasma configuration and a concentric quartz nebulizer under optimized instrumental conditions (Table S2).

### 3.5. Ozonation Tests

Ozonation experiments were conducted in a series of glass tubes (25 × 200 mm) containing 25 mL samples of NOF solution (3.31 × 10^−6^ M) and 50 mg of dry clay catalyst in a similar procedure as previously. Ozone was generated by an A_2_Z generator (A_2_Z Ozone Inc., Louisville, KY, USA) and continuously bubbled into the solution through a porous glass diffuser at a 600 mg/h throughput. Prior to UV–Vis analysis, the resulting ozonized mixtures were centrifuged and then measured using UV-Vis spectrophotometry (Agilent-Cary 60 instrument, Santa Clara, CA, USA) and 1 cm quartz cell) over the range 190–800 nm.

To complete the role of the different parameters studied previously [29,30], deeper insights in their combined effects (Interactions) on the ozonation process were achieved through a 3^3^-factorial design of experiments in agreement with previous works [59,66,67]. For this purpose, 27 ozonation tests were carried out for each catalyst, by varying the initial pH of the solution (X_1_), catalyst amount (X_2_) and ozonation time (X_3_) (Table S3). The individual effects and interactions of these parameters were assessed based on measurements of the relative absorbance of three UV-Vis key bands of NOF (Y_1_, Y_2_, Y_3_), the resulting toxicity evaluated by fresh weight, frond number, number of specimens, Chl *a/b* and ROS (Y_4_, Y_5_, Y_6_, Y_7_, Y_8_) and the final pH obtained after ozonation (Y_9_).

The instant pH was measured periodically during ozonation using an Accumet^®^ model 15 pH-meter (Fisher Scientific, Pittsburgh, PA, USA), with an accuracy of ±0.01 units. The concentration of metal cations released from the clay minerals after each ozonation test was determined using ICP-OES under the same instrumental conditions.

### 3.6. Toxicity Tests on Lemna minor

Aquatic plant *Lemna minor* was used as bioindicator for toxicity assessment, after cultivation under laboratory conditions in a nutrient medium previously prepared according to the Swedish Institute for Standards (SIS) (Table S4). A photoperiod of 16 h light and 8 h dark was maintained with light intensity maintained of 100 ± 10 µmol of photons m^−2^s^−1^ at 24 °C ± 2 °C and a relative humidity of 60 ± 5%. The cultivation medium was renewed every 7 days to ensure stable nutrient conditions and pH. The plants were exposed to *i*. NOF ozonation or adsorption mixtures in the presence of modified clay minerals and *ii*. NOF in the presence of various metal species exhibiting different oxidation states in the absence of such materials.

The number of fronds, fresh weight, the ROS production and Chlorophyll *a* fluorescence measurements were assessed through procedures fully described elsewhere [29,30]. Shortly, the plant growth inhibition was evaluated on the basis of the frond number and fresh weight. The production of reactive oxygen species (ROS) was quantified using CellROX Green, while the chlorophyll contents was determined by UV–Vis spectrophotometry after ethanol extraction.

Chlorophyll *a* fluorescence measurements were performed with Handy-PEA fluorimeter (Plant Efficiency Analyser, Hansatech Instruments Ltd., King’s Lynn, UK). This is commonly used in plant ecotoxicology to evaluate photosystem II efficiency through OJIP transient analysis and to assess the performance index (PI_ABS_) as detailed elsewhere [30,80]. The latter was determined as PI_ABS_ = RC/ABS × φPo/(1 − φPo) × ψEo/(1 − ψEo), where: 1. RC/ABS indicates the density of active reaction centers (RC) per energy dissipation from chlorophyll antenna; 2. φPo, estimated as the quantum yield of primary photochemistry, which reflects the efficiency of light energy conversion into redox energy, φPo = 1 − Fo/Fm. The Fo accounts for the fluorescence intensity at 20 µs, while Fm is the maximum fluorescence intensity; 3. ψEo is the efficiency of electron transport beyond the quinone A (Q_A_) in the PSII, derived from ψEo = 1 − [(Fj − Fo)/(Fm − Fo)]. The Fj represents the fluorescence intensity at 2 ms [81,82].

The metals concentration was determined in the testing medium and plant biomass by using the ICP-OES. Before the analysis, the digestion was first carried out in 2 mL of HNO_3_ (Fisher Scientific, purity 70%). The tubes were placed on a sand bath at 90 °C for 2 h. After digestion, each sample was collected and acidified with HNO_3_ to obtain a final concentration of 5% HNO_3_.

## 4. Conclusions

This study highlights the critical role of metal speciation and valence state in determining both the reactivity of norfloxacin in metal-loaded suspensions in water and ecotoxicological impact. NOF binds to metals through carboxyl, carbonyl, and nitrogen donor groups. Dissolved metals play a central role in the clay mineral dispersion in the aqueous media and determine its interactions with organic molecules and catalytic activity. Zero valent metal-loaded montmorillonite exhibited markedly higher toxicity, because they amplify the ecotoxicological risks through combined effects of metal release, ROS generation, and pollutant-metal complexation. Such findings provide evidence that coupling degradation studies with ecotoxicological assessments is an essential requirement when evaluating the performance of advanced clay-based treatment in the presence of metals and environmental safety.

## Data Availability

The original contributions presented in this study are included in the article/Supplementary Materials. Further inquiries can be directed to the corresponding author.

## Supplementary Materials

The following supporting information can be downloaded at: https://www.mdpi.com/article/10.3390/ijms27010459/s1.
