# Supplementary material for: Molecular Interactions of Norfloxacin in Metal-Loaded Clay Suspensions-Effects on Degradation and Induced Toxicity
Source: Int J Mol Sci. 2026 Jan 1;27(1):459. doi: 10.3390/ijms27010459 (PMC12787303; doi:10.3390/ijms27010459)
Supplement: Supplementary file 1 [file ijms-27-00459-s001.zip › ijms-4051508-supplementary.pdf]

International Journal of Molecular Sciences

Molecular Interactions of Norfloxacin in Metal-Loaded Clay Suspensions-Effects on Degradation and Induced Toxicity

Roumaissa Djidja [1], David Dewez [1,*] and Abdelkrim Azzouz [1,2,*]

1. Operating conditions for HPLC-DAD and ICP-OES measurements

Table S1. Parameters of HPLC-DAD analysis.

Parameter	Specification
Apparatus model	Agilent Technologies model 1290 equipment
Column specification	C18 column 4.6 ×150 mm,5 μm particle size
Mobile phases composition	A: water with 0.1% Formic Acid<br>B: methanol
Detection wavelength	275 nm
Gradient / isocratic	0-0.5 min, 10% B; 0.5-2.0 min, 10-15% B; 2.0-8.0 min, 15-35% B; 8.0-10.0 min, 35-90% B; 10.0-11.0 min, 90% B; 11.0-11.2 min, 90-10% B; 11.2-18.0 min, 10%B.
Flow rate	1 mL. min^{-1}
Temperature	20 °C
Pump pressure	18 MPa
Injected volume	20 uL

Table S2. Instrumental parameters of ICP-OES.

Parameter	value
Power	1.5 kW
Plasma argon flow rate	12 L/min
Auxiliary argon flow rate	1 L/min
Nebulizer argon flow rate	1 L/min
Wavelengths (nm)	Na (589.592), Fe (238.204), Ni (216.555), Cu (199.970) and Co (228.615).

2. Factorial design for the 27 NOF ozonation attempts

Table S3. Parameter variation ranges for 3^2 factorial designs at T = 25 °C.

Parameter	Symbol (Coded Variation)	Variation levels [a]		
		Minimum (-1)	Center (0)	Maximum (+1)
pH	X_1	3.2	4.2	5.2
Clay material amount (g. L^{-1})	X_2	1	2	3
Ozonation time (min)	X_3	1	5	9

[a] In brackets: the coded value of the variation levels for expressing the reduced (non-dimensional) value of each parameter.

 https://doi.org/10.3390/ijms27010459

3. SIS media preparation

Table S4. Preparation and composition according to Swedish Standard for *Lemna* growth medium (SIS).

Stock solution	Chemicals	Concentration in		Manufacturer
		Stock solution (g/L)	SIS medium* (mg/L)	
I	Na NO_3	8.500	85.000	Anachemia, ACS grade
	KH_2PO_4	1.340	13.400	Anachemia
II	$MgSO_4.7H_2O$	15.000	75.000	Anachemia
III	$CaCl_2.2H_2O$	7.200	36.000	Anachemia, ACS grade
IV	Na_2CO_3	4.000	20.000	Fisher Scientific
V	$ZnSO_4.7H_2O$	0.050	0.050	Anachemia, ACS grade
	H_3BO_3	1.000	1.000	Anachemia, ACS grade
	$MnCl_2.4H_2O$	0.200	0.200	Anachemia, ACS grade
	$Na_2MoO_4.2H_2O$	0.010	0.010	Fisher Chemicals
	CuSO4.5H2O	0.005	0.005	Anachemia, ACS grade
	$Co(NO_3).6H_2O$	0.010	0.010	Anachemia, ACS grade
VI	$FeCl_3.6H_2O$	0.170	0.840	Anachemia
	Na_2-EDTA.$2H_2O$	0.280	1.400	Anachemia
VII	MOPS (buffer)	490.000	490.000	Anachemia

* The SIS medium was prepared through the dissolution of 10 mL of solution I, 5 mL of solution II, 5 mL of solution III, 5 mL of solution IV, 1 mL of solution V, 5 mL of solution VI, 1 mL of solution VII in a 1 L flask containing ca 900 mL of ultrapure water and dissolution of the following stock solutions. The pH was adjusted to 6.5 ± 0.2 with either 0.1 M HCl or 0.1 M NaOH. The volume was finally adjusted to 1 litre.

4. Metal cation:NOF interaction

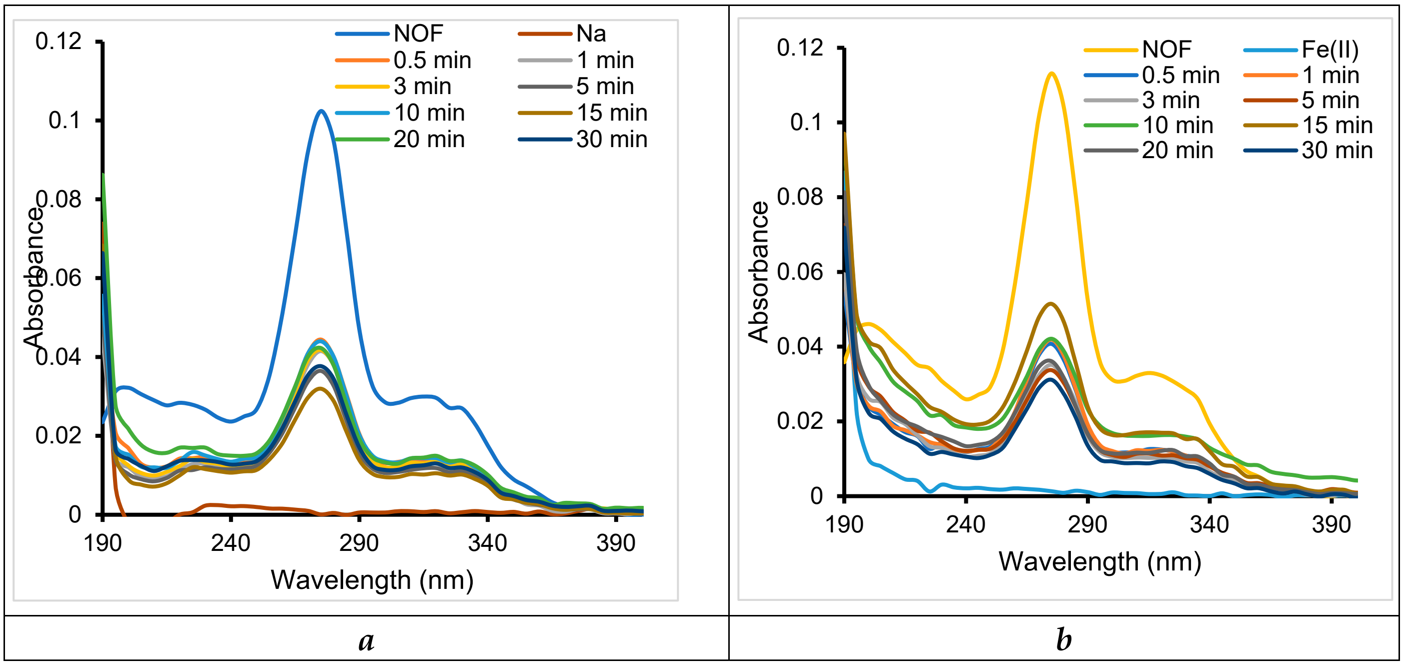

a	*b*

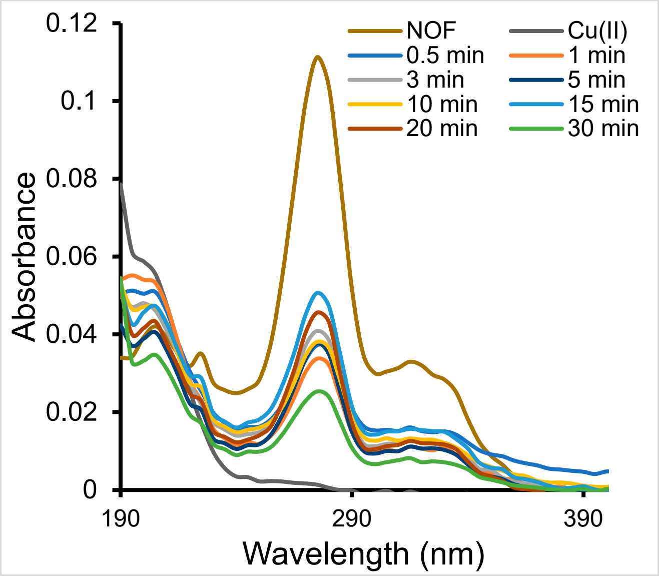

c

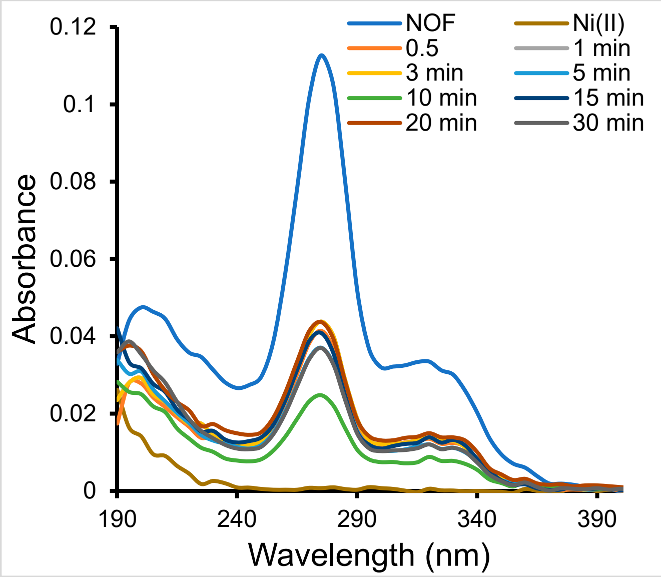

d

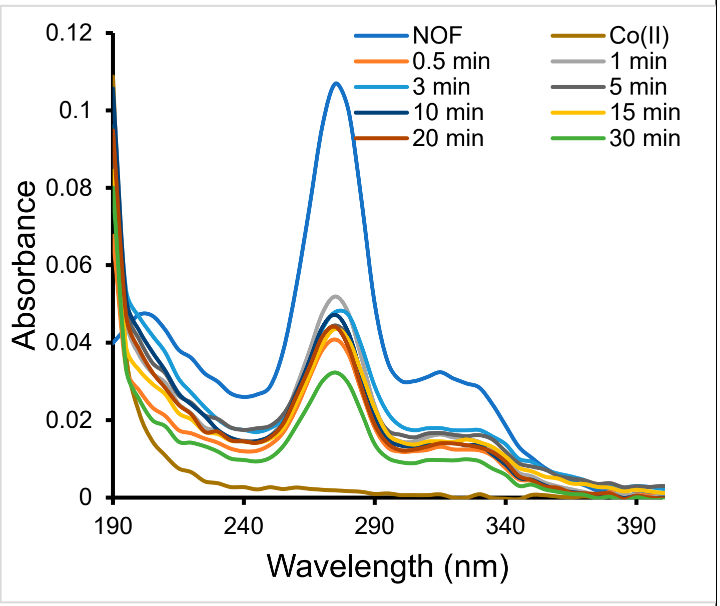

e

Figure S1. UV–vis absorption spectra for Norfloxacin, metal ions and their complexes at different contact time. Na (a), Fe (b), Cu (c), Ni (d) and Co (e)

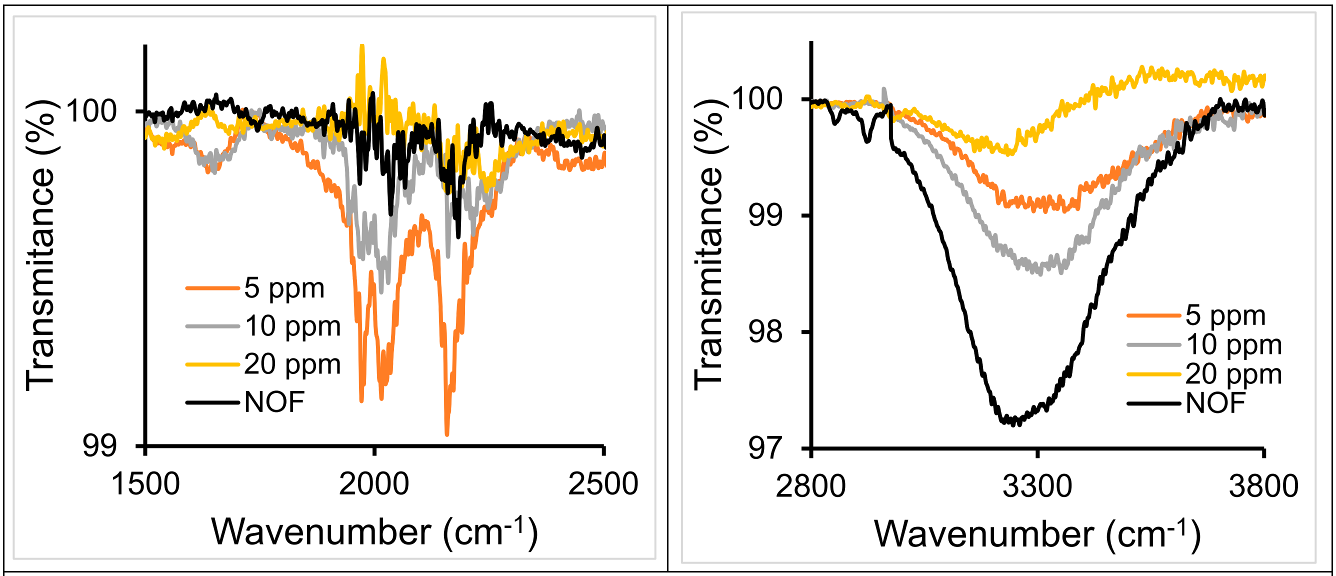
Transmitance (%)
Wavenumber (cm-1)
5 ppm
10 ppm
20 ppm
NOF

A

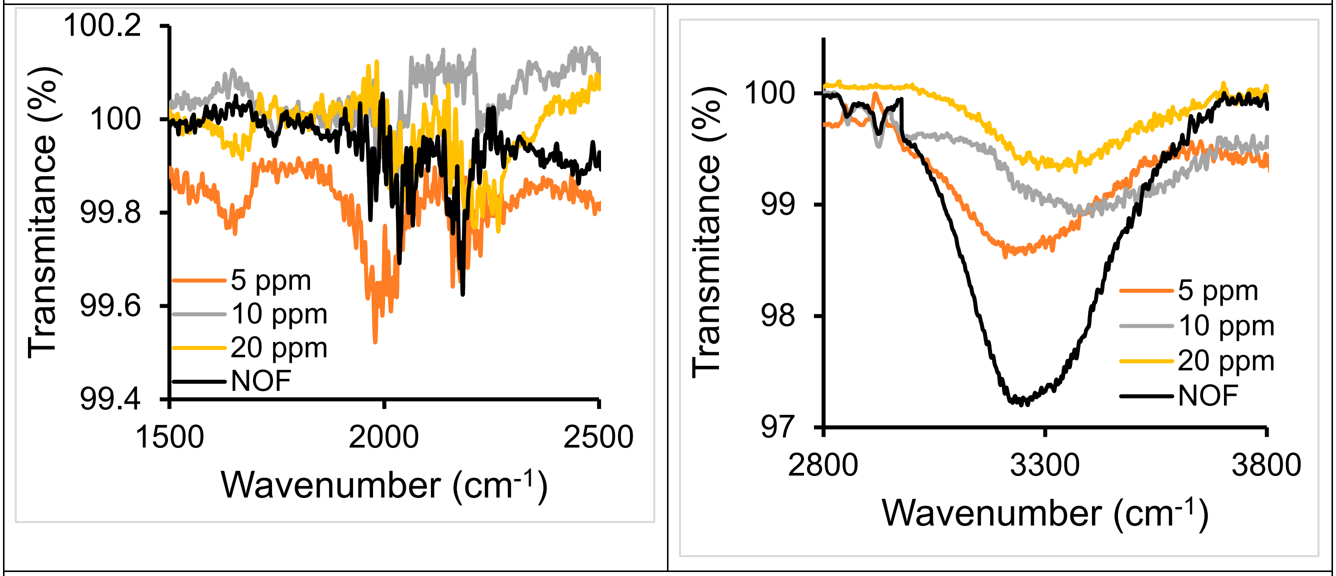
Transmitance (%)
Wavenumber (cm-1)
5 ppm
10 ppm
20 ppm
NOF

b

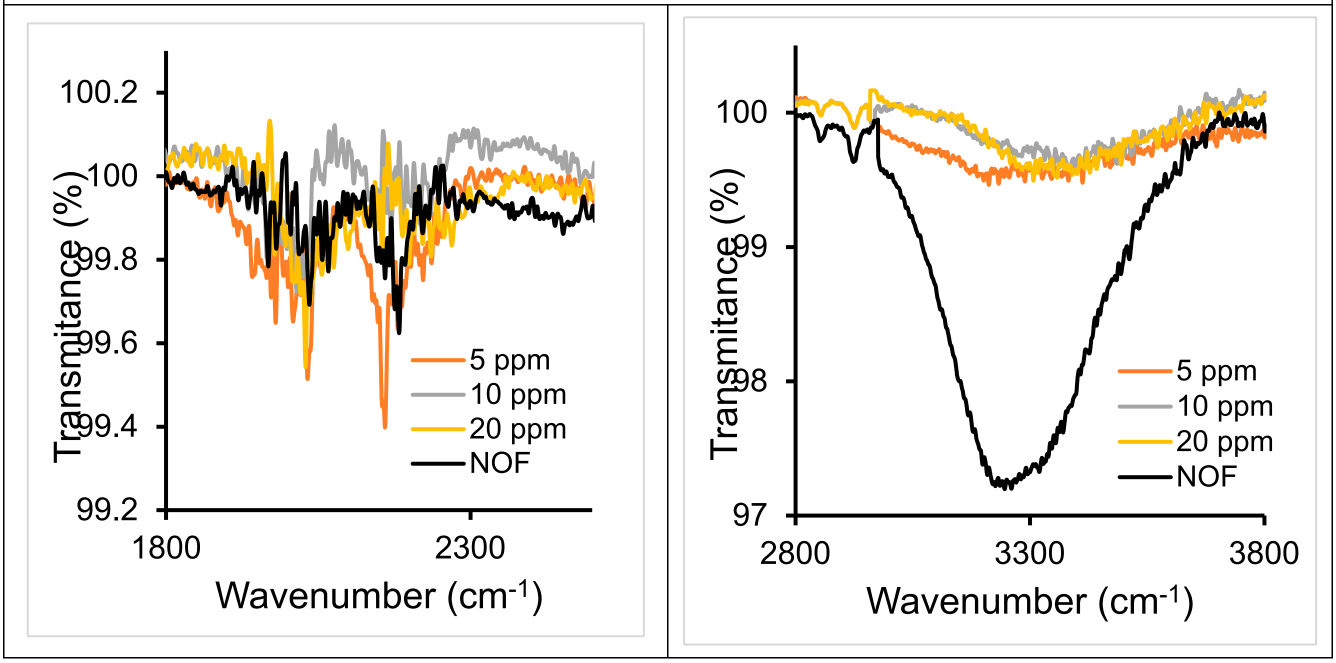
Transmitance (%)
Wavenumber (cm-1)
5 ppm
10 ppm
20 ppm
NOF

c

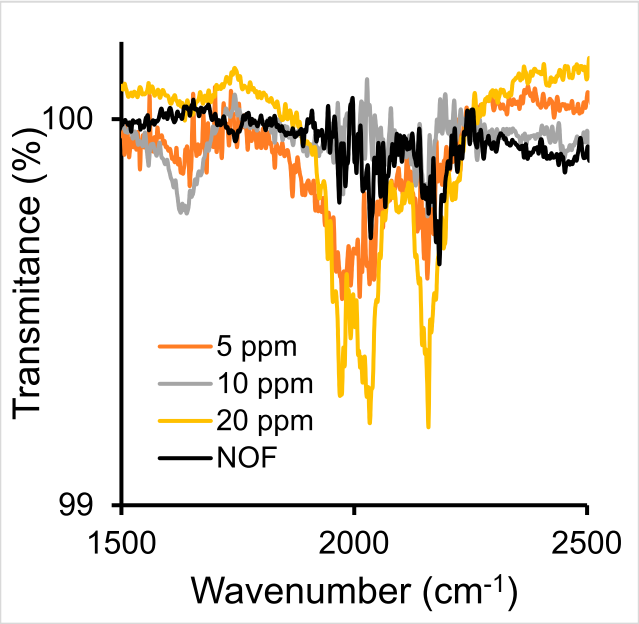

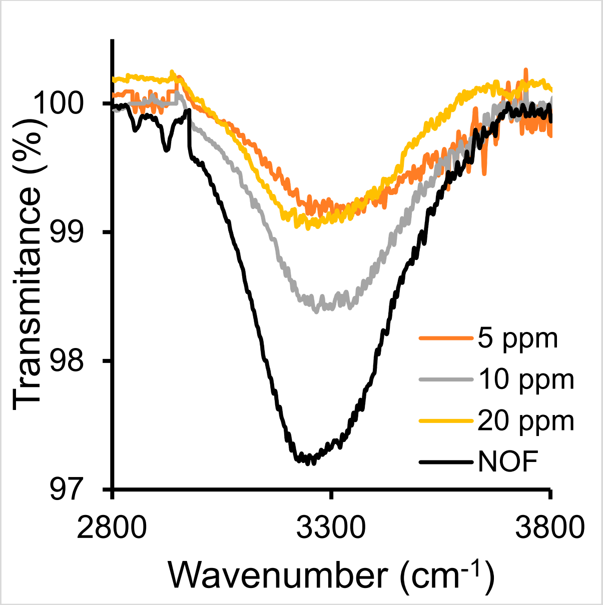

d

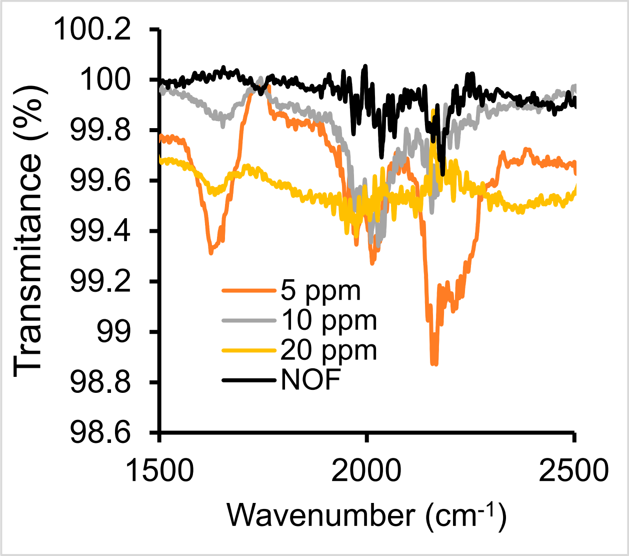

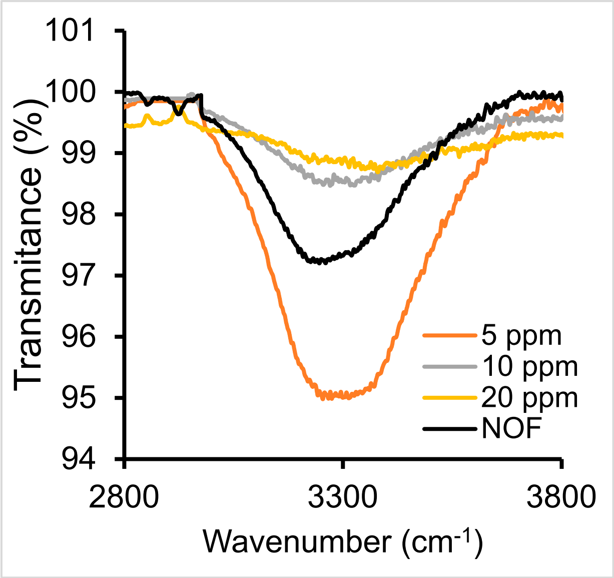

e

Figure S2. Effect of the concentration of Na^{+} (a), Fe^{2+} (b), Cu^{2+} (c), Ni^{2+} (d) or Co^{2+} cation (e) on NOF solution IR spectrum: NOF conc.: 3.31×10^{-6} M.

Table S5. FTIR vibrational changes of norfloxacin upon metal addition.

Suspected interaction	Conc. (ppm)	N-H stretching O-H stretching	C=O stretching	N-H bending
		Wavenumber (cm^{-1})		
NOF	1	3260	1740	-
[NOF:Na^{+}]	5	3320	-	1650
	10	3320	-	1650
	20	3270	-	-
[NOF:Fe^{2+}]	5	3240	-	1650
	10	3420	-	1670

	20	3370	-	1670
$[NOF:Cu^{2+}]$	5	3280	-	-
	10	3380	-	1650
	20	3380	-	1650
$[NOF:Ni^{2+}]$	5	3320	-	1660
	10	3300	-	1640
	20	3280	-	-
$[NOF:Co^{2+}]$	5	3290	-	1620
	10	3300	-	1650
	20	3320	-	1630

5. Parameter interaction and effect on NOF degradation and toxicity

Table S6. NOF relative absorbance ($Y_{1,2,3}$) for 200, 275, 315 nm respectively, fresh wight (Y_4), fronds number (Y_5), number of plants (Y_6), Chl *a*/*b* (Y_7), ROS (Y_8) and final pH measurements (Y_9) for NaMt and Fe(II)Mt catalyst system.

					NaMt					
X_1	X_2	X_3	A/A_{0_200} Y_1	A/A_{0_275} Y_2	A/A_{0_315}	Fresh weight Y_4	Fronds numbe Y_5	Number of plants Y_6	Chl *a*/*b* Y_7	ROS Y_8
-1	-1	-1	17,9491	1,6233	1,9700	0.098	54	15	0,8838	168,9828
-1	-1	0	35.8687	1.7021	2.0211	0.079	50	10	0.8838	168.9828
-1	-1	1	45.0644	1.5136	1.8097	0.009	9	9	0.4184	321,9287
-1	0	-1	24.3342	2.3581	2.8636	0.090	46	8	1,2682	646,8543
-1	0	0	46.9685	2.8231	3.3335	0.073	47	15	1,2431	1237,4170
-1	0	1	9.3204	0.6182	1.5317	0.082	44	12	1,3142	423,5099
-1	1	-1	23.9497	2.5182	2.9579	0.065	38	12	1,3423	644,0141
-1	1	0	41.4197	3.5855	4.4993	0.055	30	11	1,2992	1977,4650
-1	1	1	16,4598	1,0420	2,5281	0.064	33	7	1,2787	349,2958
0	-1	-1	13,2365	1,1648	1,4708	0.075	50	12	1,2358	6,9549
0	-1	0	40.2056	1.4840	1.9228	0.064	46	10	1.2183	242,1321
0	-1	1	45,2579	1,0638	1,1678	0.048	40	9	1,2474	103,8668
0	0	-1	33,2118	3,6164	3,5118	0.060	33	10	1,2648	765,3496
0	0	0	39.1972	2.2873	2.5990	0.069	48	11	1.0875	83,6273
0	0	0	41.1368	2.0928	2.3546	0.087	45	13	1.0875	84.2756
0	0	0	40.4841	2.0884	2.4649	0.084	53	13	1.0778	86,6549
0	0	1	53.3965	2.7666	3.2864	0.066	40	9	1.2992	316,8693
0	1	-1	28.3953	2.7059	3.2392	0.065	44	11	1.4345	548,6280
0	1	0	45.4518	4.7815	4.6840	0.078	40	11	1.4062	781,5549
0	1	1	14,7772	0,9314	2,1056	0.061	36	10	1,3704	1366,3110
1	-1	-1	20.8299	1.4111	1.7579	0.038	28	8	1.1553	418,3673
1	-1	0	40.4083	1.7101	2.0554	0.061	32	10	1.3757	81,2777
1	-1	1	50.9514	3.0569	3.4698	0.080	56	17	1.6233	287,0452
1	0	-1	43.3925	2.5703	3.5222	0.019	23	9	1.2079	85,4326
1	0	0	40.7216	2.4962	2.9241	0.042	28	9	1.4310	258,5980
1	0	1	44.2105	1.7574	2.1017	0.063	28	9	1.3288	79,8832
1	1	-1	35.9819	5.0071	5.0071	0.076	36	11	1.5347	57,6768
1	1	0	45.1411	4.6585	4.6584	0.064	33	11	1.3843	194,2424
1	1	1	51,2096	4,3533	4,3533	0.030	26	9	1,2544	591,4647
					Fe(II)Mt					
-1	-1	-1	7.4360	0.3324	0.9358	0.085	55	17	1.7016	474.2496

-1	-1	0	29.9904	0.3468	0.9770	0.032	43	12	1.4882	621.9589
-1	-1	1	41.2761	0.2928	0.8233	0.001	9	9	0.4960	572.9589
-1	0	-1	7.8839	0.3037	0.8322	0.099	65	23	1.6880	3.3592
-1	0	0	27,9383	0,3663	0,9991	0.029	35	11	1,6165	16,3204
-1	0	1	39.7397	0.3122	0.8595	0.003	9	9	0.5996	0.2187
-1	1	-1	8.8689	0.3326	0.8697	0.086	57	17	1.6784	1723.8727
-1	1	0	27,3644	0,3278	0,9095	0.018	27	8	1,3579	3073,2091
-1	1	1	45.9466	0.4112	1.1190	0.004	12	12	0.5132	2557.0292
0	-1	-1	8.01741	0.5565	1.5360	0.092	64	20	1.1118	143,1301
0	-1	0	29,2932	0,5178	1,4731	0.099	71	22	1,3104	46,0163
0	-1	1	29.3615	0.3247	0.8827	0.001	9	9	0.3922	286,9106
0	0	-1	7.5331	0.5493	1.4338	0.088	57	18	0.8776	1866.9064
0	0	0	26.9505	0.5422	1.4189	0.088	74	23	1.0862	1269,7842
0	0	0	26,2587	0.5391	1.3974	0.083	69	21	1,04840	1264.5458
0	0	0	25.9573	0.5179	1.3806	0.086	71	21	1,07985	1262.3795
0	0	1	40.3938	0.5776	1.5277	0.002	9	9	0.4840	906,4748
0	1	-1	7.6516	0.6171	1.5684	0.088	56	18	1.1273	3,5235
0	1	0	24.8021	0.6307	1.6019	0.083	62	21	1.1531	32,1216
0	1	1	36.8586	0.4700	1.2020	0.028	24	9	0.8301	151.8456
1	-1	-1	8,64501	0,4925	1,3279	0.113	68	24	0,0794	182,4687
1	-1	0	25.0314	0.5158	1.4389	0.081	63	21	1.2131	843.8283
1	-1	1	37,2302	0,5189	1,4481	0.006	11	11	0,0914	293,3810
1	0	-1	8,46069	0,5748	1,5394	0.103	68	22	1,3212	3076,6917
1	0	0	27.1959	0.6301	1.6326	0.073	62	19	1.2267	3485.7143
1	0	1	32.3008	0.5224	1.3610	0.003	8	8	0.7668	6022,5564
1	1	-1	7,82108	0,6092	1,5242	0.096	62	20	1,0088	313,3735
1	1	0	25.0325	0.6519	1.6417	0.093	64	21	1.0593	336.5060
1	1	1	35,3533	0,3755	0,9256	0.006	9	9	0,3957	985,3012

Table S7. Polynomial models for the response-functions in NOF ozonation with NaMt and Fe(II)Mt.

Catalyst	Response-function		Polynomial coefficients										
			a_0	a_1 pH effect	a_2 clay amount effect	a_3 ozonation time effect	a_{11}	a_{22}	a_{33}	a_{12}	a_{13}	a_{23}	a_{123}
NaMt	A/A_0 (200nm)	Y_1	43,525	6,195	-0,388	4,965	0,440	-3,164	-9,94	3,10	3,463	-7,928	2,464
	A/A_0 (275nm)	Y_2	2,654	0,513	0,825	-0,326	0,178	0,096	-0,61	0,461	0,292	-0,445	-0,117
	A/A_0 (315nm)	Y_3	2,977	0,352	0,910	-0,219	0,299	0,018	-0,49	0,213	0,130	-0,289	-0,262
	Fresh weight	Y_4	0,069	-0,008	0,000	-0,005	-0,005	-0,001	0,004	0,005	-0,001	-0,004	-0,022

	Frond number	Y_5	43,259	-3,389	-2,722	-2,222	-6,278	0,389	-2,44	-0,75	6,250	0,333	-9,750
	Number of plants	Y_6	10,333	-0,333	-0,389	-0,278	0,333	0,500	-0,50	0,000	1,167	-0,667	-1,500
	Clh a/b	Y_7	1,312	0,132	0,126	-0,011	-0,050	-0,030	-0,01	-0,14	0,066	-0,035	-0,144
	ROS	Y_8	148,901	-216,9	260,54	27,66	-23,15	29,806	-162	-186	63,503	78,185	139,097
Fe(II)Mt	A/A_0 (200nm)	Y_1	26,290	-1,63	0,190	14,786	1,211	-0,05	-4,24	-0,5	-1,90	0,837	-0,536
	A/A_0 (275nm)	Y_2	0,581	0,104	0,029	-0,031	-0,092	-0,024	-0,05	0,001	-0,026	-0,005	-0,047
	A/A_0 (315nm)	Y_3	1,534	0,251	0,029	-0,079	-0,229	-0,056	-0,14	-0,02	-0,068	-0,006	-0,135
	Fresh weight	Y_4	0,073	0,012	0,000	-0,044	-0,012	0,002	-0,02	0,000	-0,003	0,004	0,002
	Frond number	Y_5	60,593	5,722	-1,111	-25,11	-6,944	-0,444	-19,4	0,333	-1,917	2,333	0,375
	Number of plants	Y_6	18,630	2,056	-0,556	-5,222	-1,389	-0,222	-2,89	-0,42	-0,917	0,583 inf errer	-0,125
	Clh a/b	Y_7	1,308	-0,221	0,069	-0,335	0,086	-0,129	-0,44	0,101	0,192	-0,014	-0,083
	ROS	Y_8	1283,804	360,967	317,369	221,574	842,712	-1147	6,254	-448	233,395	108,397	-21,774

Table S8. Calculated adimensional/real optimum parameters for catalytic ozonation.

Response function		value	NaMt			Fe(II)Mt		
			X_1	X_2	X_3	X_1	X_2	X_2
A/A_0 (200nm)	Y1	Adimensional	-	0.555	0.471	-	-17.665	0
			-11.7	-	-1.789	0.749	-	0.096
			-4.079	-1.998	-	0	-3.169	-
		Real (g.L^{-1}; min)	-	63.9	7	-	492	5
			16	-	12	4.9	-	5
			8.3	99	-	4.2	129	-
A/A_0 (275nm)	Y2	Adimensional	-	-2.664	0.705	-	0.604	-0.316
			-1.021	-	-0.512	0.634	-	-0.484
			-1.955	0.397	-	0.565	0.604	-
		Real (g. L^{-1}; min)	-	117	8	-	65	6
			5.2	-	7	4.8	-	7
			6.2	60	-	4.8	65	-
A/A_0 (315nm)	Y3	Adimensional	-	11.348	3.149	-	0.259	-0.288
			-0.524	-	-0.295	0.613	-	-0.441
			-4.27	10.342	-	0.541	0.143	-

		Real (g. L^{-1}; min)	-	334	18	-	56	6
			4.7	-	6	4.8	-	7
			8.5	308	-	4.7	54	-
Fresh weight	Y4	Adimensional	-	0	0	-	11	-11
			0	-	0	-14.667	-	121.333
			0	0	-	0	0.5	-
		Real (g. L^{-1}; min)	-	50	5	-	325	49
			4.2	-	5	18.9	-	489
			4.2	50	-	4.2	63	-
Frond number	Y5	Adimensional	-	0	0	-	10.763	0
			0	-	0.542	0.412	-	-0.646
			-0.27	0	-	0.412	0	-
		Real (g. L^{-1}; min)	-	50	5	-	319	5
			4.2	-	5	4.6	-	8
			4.5	50	-	4.6	50	-
Number of plants	Y6	Adimensional	-	0	0	-	0	-0.301
			0	-	0	0.74	-	-0.904
			0	0	-	0.74	0	-
		Real (g. L^{-1}; min)	-	50	5	-	50	6
			4.2	-	5	4.9	-	9
			4.2	50	-	4.9	50	-
Chl *a*/*b*	Y7	Adimensional	-	0	3.6	-	0.267	-0.383
			0	-	-2	1.375	-	-0.081
			0.699	0.434	-	0.017	0.626	-
		Real (g. L^{-1}; min)	-	50	19	-	57	7
			4.2	-	13	5.6	-	5
			4.9	61	-	5.1	66	-
ROS	Y8	Adimensional	-	-3.404	-0.737	-	-0.496	-13.42
			6.573	-	1.376	-1.413	-	8.657
			0.951	-1.403	-	-0.169	0.171	-
		Real (g. L^{-1}; min)	-	135	8	-	62	59
			10.8	-	11	5.6	-	40
			5.2	85	-	4.4	54	-

* Certain values are outside the considered range and were not considered for the determination of optimum response-functions. They are qualitatively assessed using a formula that is valid only within the considered parameter ranges.

6. Ozonation effect on photosynthetical system

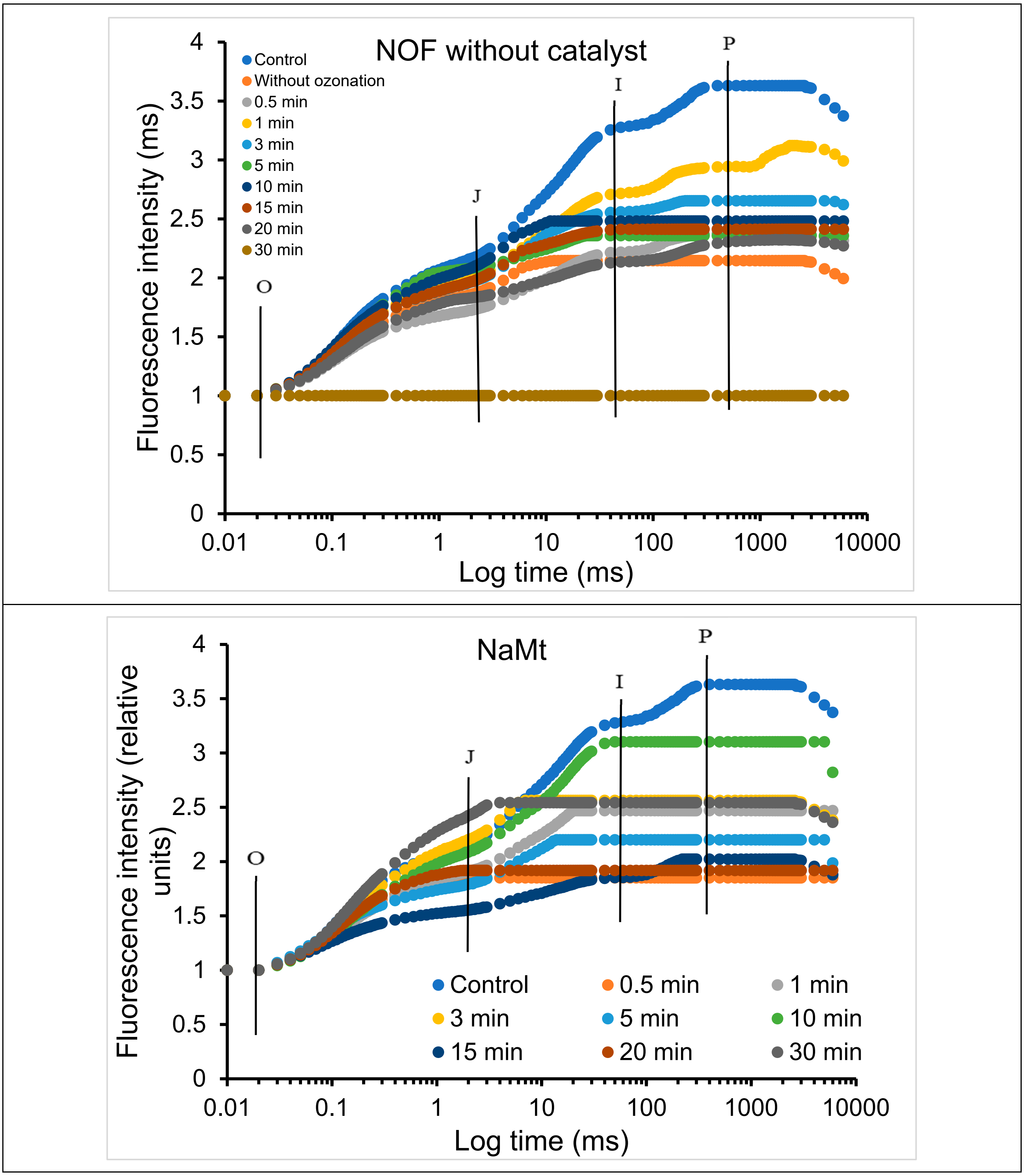
NOF without catalyst
Control
Without ozonation
0.5 min
1 min
3 min
5 min
10 min
15 min
20 min
30 min
O
J
I
P
Fluorescence intensity (ms)
Log time (ms)
0.01
0.1
1
10
100
1000
10000
NaMt
Control
0.5 min
1 min
3 min
5 min
10 min
15 min
20 min
30 min
Fluorescence intensity (relative units)
Log time (ms)

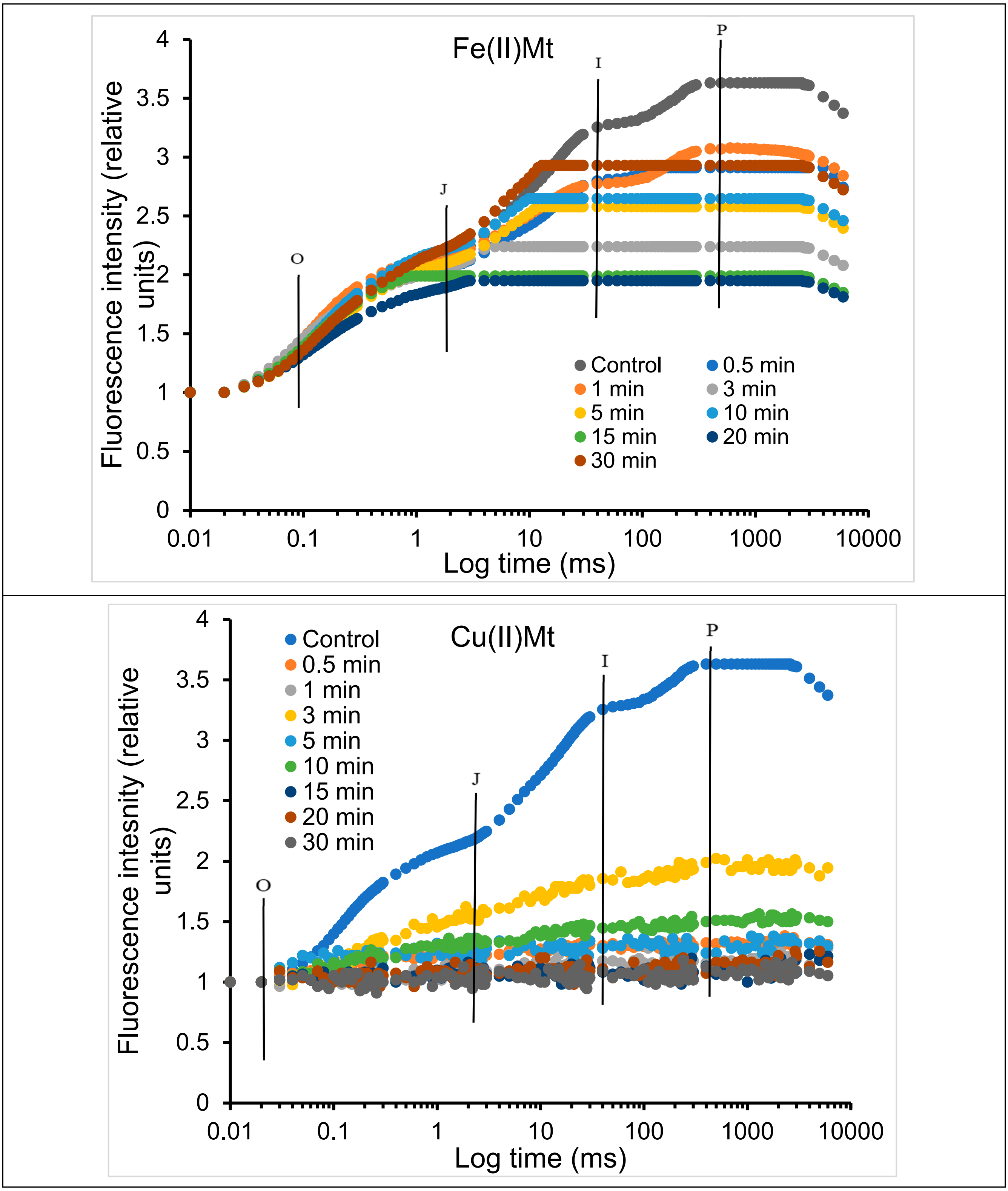

Fe(II)Mt
O
J
I
P
Control
0.5 min
1 min
3 min
5 min
10 min
15 min
20 min
30 min
Fluorescence intensity (relative units)
Log time (ms)
0
0.5
1
1.5
2
2.5
3
3.5
4
0.01
0.1
1
10
100
1000
10000
Cu(II)Mt
O
J
I
P
Control
0.5 min
1 min
3 min
5 min
10 min
15 min
20 min
30 min
Fluorescence intesnity (relative units)
Log time (ms)
0
0.5
1
1.5
2
2.5
3
3.5
4
0.01
0.1
1
10
100
1000
10000

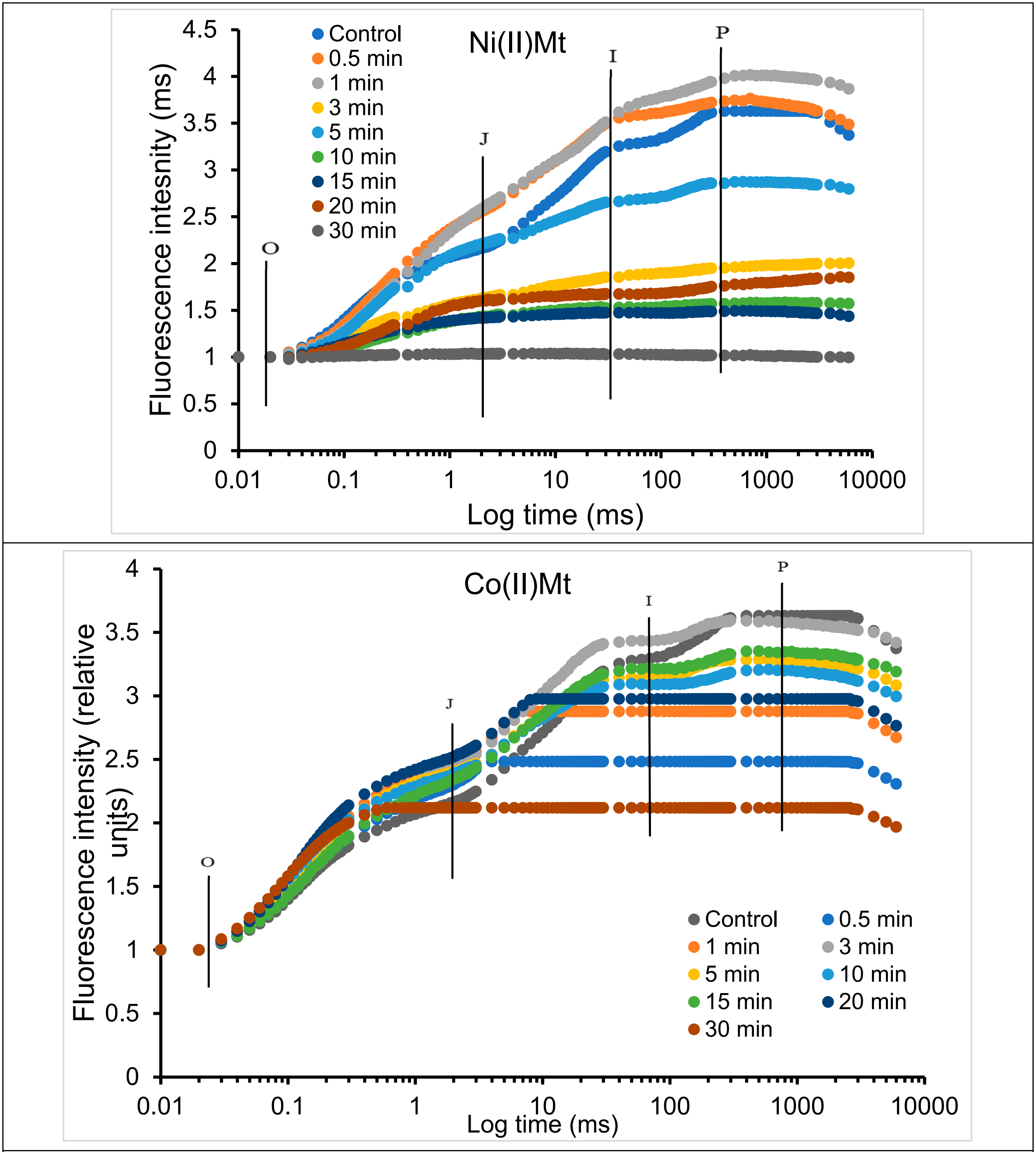

Figure S3. Change in the fast and polyphasic fluorescence kinetics of chlorophyll *a* of *L. minor*. Plants were exposed for 7 days to different ozonised mixture with clay-catalyst. Symbols O, J, I and P represent, respectively, the fluorescence intensities at 50 μs, 2ms, 30ms, and 200ms.

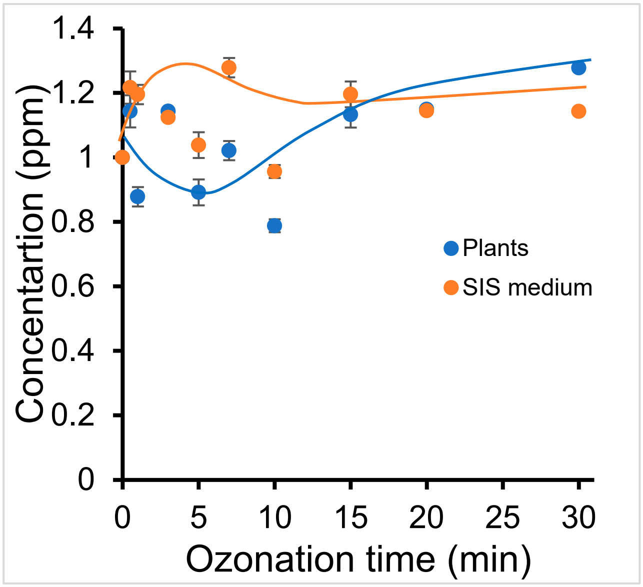
Concentartion (ppm)
Ozonation time (min)
Plants
SIS medium

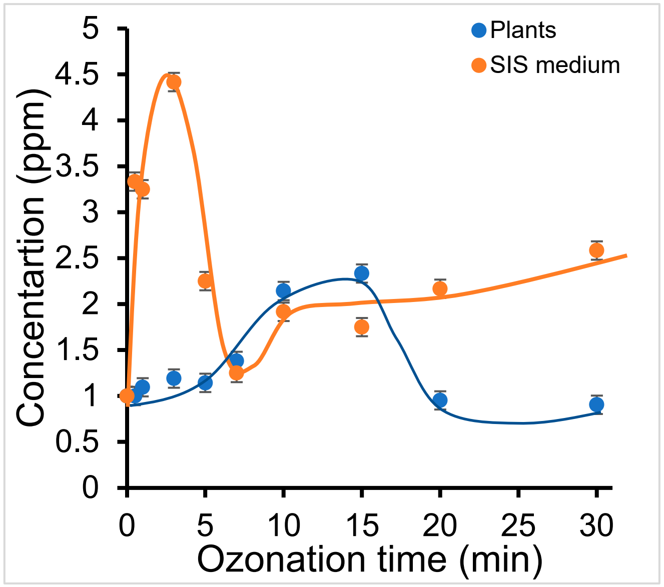
Concentartion (ppm)
Ozonation time (min)
Plants
SIS medium

a *b*

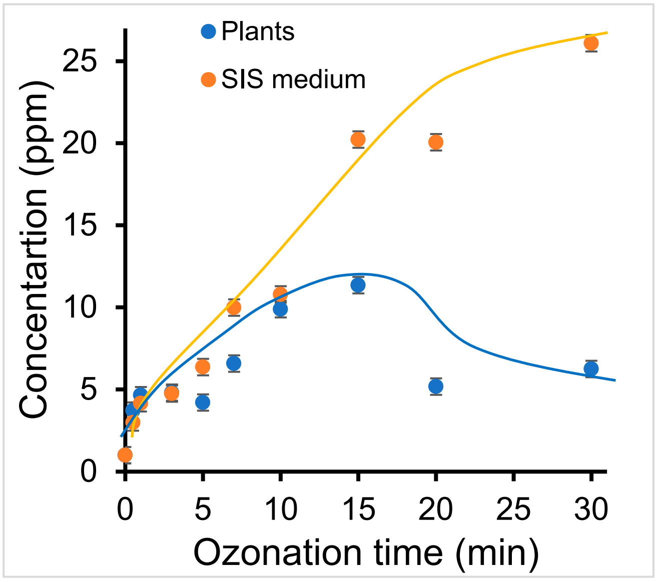
Concentartion (ppm)
Ozonation time (min)
Plants
SIS medium

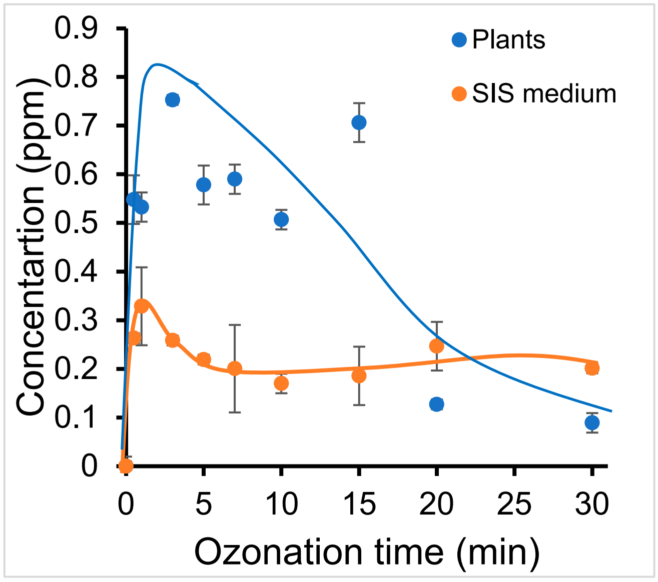
Concentartion (ppm)
Ozonation time (min)
Plants
SIS medium

c *d*

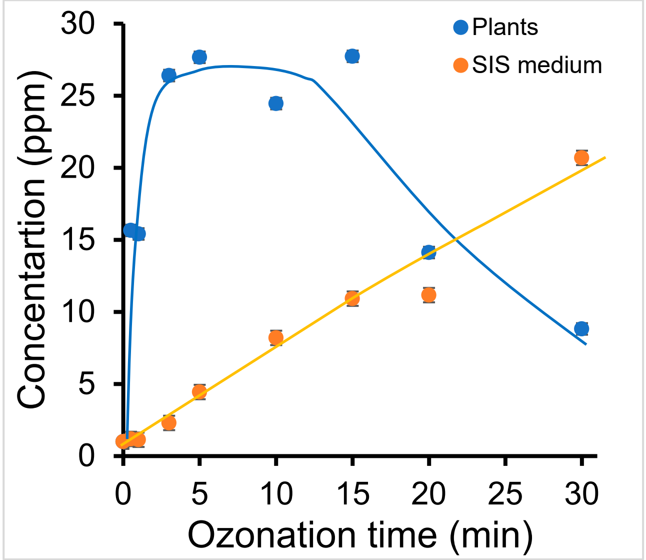
Concentartion (ppm)
Ozonation time (min)
Plants
SIS medium

e	

Figure S4. Residual metal concentrations in SIS Medium and *Lemna minor* measured by ICP-OES. Na^{+} (a), Fe^{2+} (b), Cu^{2+} (c), Ni^{2+} (d) or Co^{2+} cation (e). T = 25 ºC. pH = 5.2. Ozone flow rate = 600 mg/h. Catalyst amount: 50 mg. Sample volume: 25 mL. NOF initial concentration = 3.31×10^{-6} M. Most curves starts from the unity on the Y-axis, because, except for nickel, the concentration values were normalized to the control sample consisting of SIS medium without NOF, which naturally contains sodium, iron, copper and cobalt as a nutrient. Nickel concentrations were not normalized due to the absence of Ni in the SIS medium.

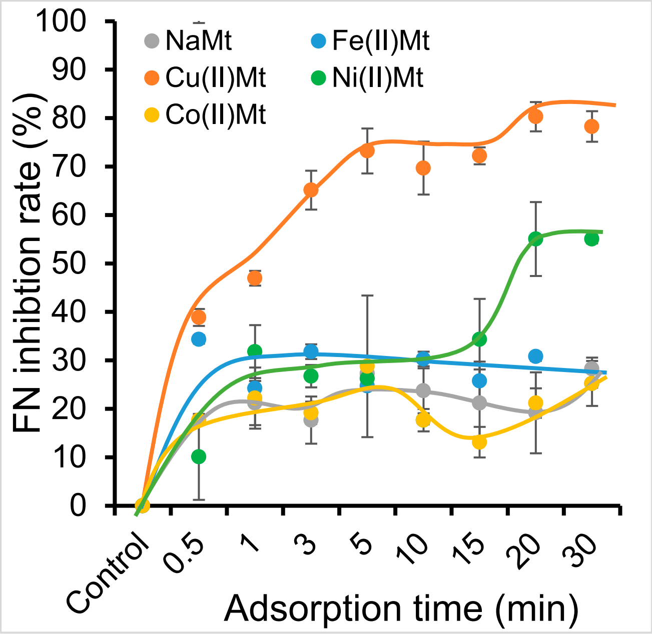

a

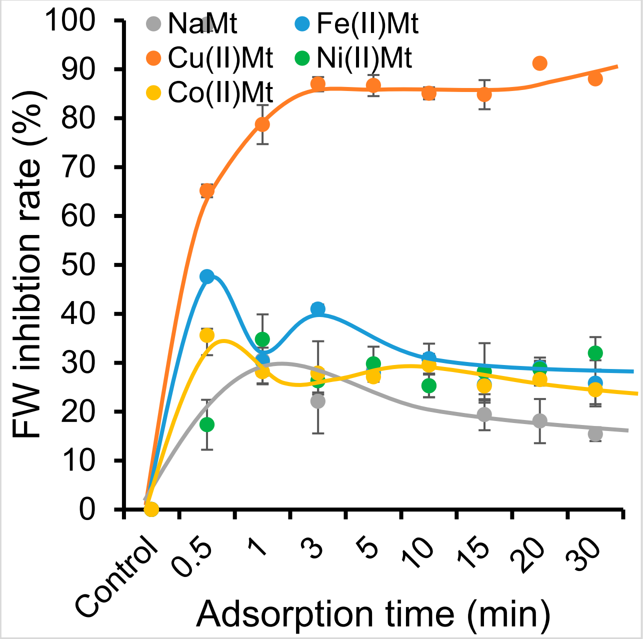

b

Figure S5. Effect of combined NOF and metal ion on growth inhibition expressed in terms of frond number (a) and fresh weight after 7 days of exposure to *Lemna minor*. Triplicates measurements were achieved including the control sample. The control sample consisted of plants grown in SIS medium.

7. ZVM-loaded Mt analysis by FTIR

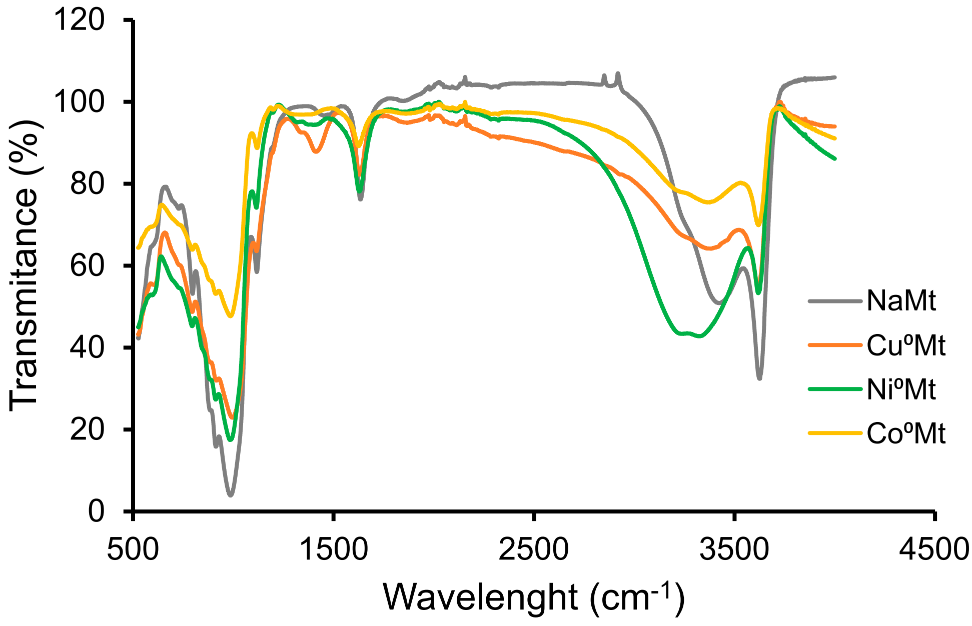

Figure S6. IR spectra of NOF-free aqueous suspensions of ZVM-montmorillonites.

8. Toxicity of NOF adsorbed on ZVM

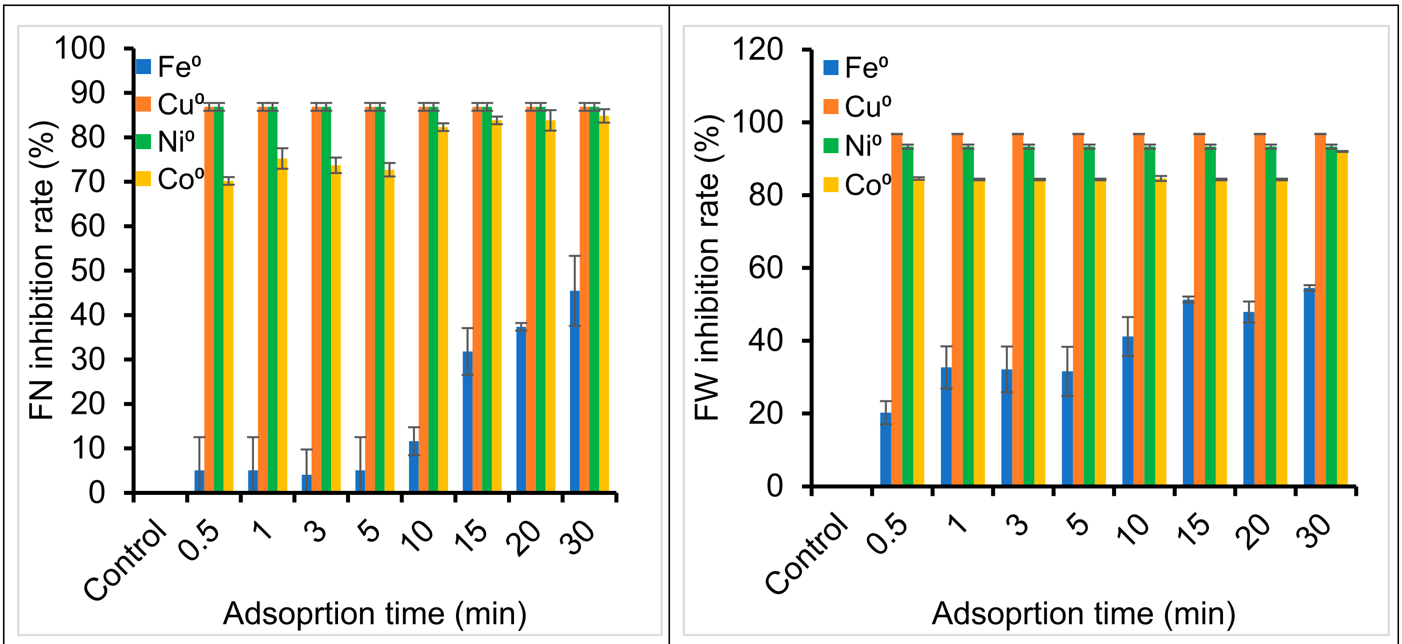

a *b*

Figure S7. Effect NOF adsorption in clay-supported ZVM on growth inhibition expressed in terms of frond number (a) and fresh weight after 7 days of exposure to *Lemna minor*. Triplicates measurements were achieved including the control sample. The control sample consisted of plants grown in SIS medium.
